# A Pilot Application of an iTRAQ-Based Proteomics Screen Estimates the Effects of Cigarette Smokers’ Serum on RPE Cells With AMD High-Risk Alleles

**DOI:** 10.1167/tvst.11.2.15

**Published:** 2022-02-09

**Authors:** Bincui Cai, Zhe Zhang, Shuo Sun, Tingting Lin, Yifeng Ke, Zhiqing Li, Jin Yang, Xiaorong Li

**Affiliations:** 1 Tianjin Key Laboratory of Retinal Functions and Diseases, Tianjin International Joint Research and Development Center of Ophthalmology and Vision Science, Eye Institute and School of Optometry, Tianjin Medical University Eye Hospital, People's Republic of China; 2 Eye Institute and Department of Ophthalmology, Eye & ENT Hospital, Fudan University, NHC Key Laboratory of Myopia (Fudan University); Key Laboratory of Myopia, Chinese Academy of Medical Sciences, Shanghai Research Center of Ophthalmology and Optometry, Shanghai, China

**Keywords:** age-related macular degeneration, cigarette smoking, iTRAQ proteomics, ARMS2, HTRA1

## Abstract

**Purpose:**

The aim of this study was to explore whether there are interactions between genetic (ARMS2/HTRA1) and environmental factors (cigarette smoking) in the pathogenesis of age-related macular degeneration (AMD).

**Methods:**

Primary human retinal pigment epithelial (hRPE) cells were obtained from four donors’ eyes with AMD high-risk ARMS2/HTRA1 alleles, and two donors’ eyes with wild-type alleles were used as controls. The pooled serum from 32 smokers and 35 nonsmokers were collected and used separately to treat hRPE cells. The isobaric tag for relative and absolute quantitation (iTRAQ)-based proteomics was used to identify associated proteins and comparing the differences between AMD high-risk and low-risk HTRA1/ARMS2 alleles after exposure to smokers’ serum.

**Results:**

After stimulation with the smokers’ serum, 400 differentially expressed proteins (DEPs) were detected in the high-risk allele cells. Several DEPs are involved in neuronal protein degeneration and oxidative stress pathways. The smokers’ serum stimulation or HTRA1 overexpression can both upregulate caveolin-1, which was one of the DEPs. Besides, the smokers’ serum enhanced the phagocytosis of cultured human RPE cells.

**Conclusions:**

The study confirmed the AMD high-risk alleles, HTRA1, and cigarette smoking can promote AMD development by regulating caveolin-1 expression.

**Translational Relevance:**

AMD high-risk alleles and environmental risk factors can promote the occurrence and development of AMD by regulating caveolin-1 expression, upregulation of which will induce apoptotic cell death in response to cellular stress in early AMD conditions.

## Introduction

Age-related macular degeneration (AMD) is a common, chronic, progressive macular degeneration disease that affects the elderly, characterized by the appearance of drusen in the macula, accompanied by choroidal neovascularization (CNV) or geographic atrophy.^1^ Current studies indicate that cigarette smoking is the only definite environmental risk factor for the development of AMD,^2^^,^^3^ and the ARMS2/HTRA1 risk alleles are considered to be high-risk genetic factors for AMD. However, whether there are any relations or interactions between genetic factors and cigarette smoking in the occurrence and development of AMD is unclear and controversial. Previous research on cigarette smoking and disease susceptibility in AMD high-risk genotype patients is limited to clinical comparative studies.^4^^,^^5^ Nakayama used transgenic (Tg) mice to determine the function of ARMS2 and HTRA1 in the choroid and retina and further evaluated the effects of mainstream cigarette smoke on these mice.^6^ However, thus far, there was very few direct experimental evidence with human samples. Here, we exposed allele-specific, autopsied retinal pigment epithelial (RPE) cells to the serum of smokers and nonsmokers to study the effects of cigarette smoking on patients with ARMS2/HTRA1 risk alleles using an isobaric tag for relative and absolute quantitation (iTRAQ)-based proteomics screen.

## Materials and Methods

### Cell Culture System

Primary human retinal pigment epithelial (hRPE) cells were isolated from autopsied eyeballs obtained from eye bank donors. The eye donors were between the ages of 35 and 67 years without any retinal diseases recorded (Table 1). The process was approved by the Ethics Committee of Tianjin Medical University Eye Hospital (Tianjin, China). The eyes were processed with the following procedures within 16 hours after being obtained. In brief, whole eyeballs were rinsed in phosphate-buffered saline (PBS) with antibiotics. The anterior segment and neural retina were removed, exposing the posterior segment. The RPE-choroid was separated from the sclera, rinsed with Hank's Balanced Salt Solution, and then treated with 0.25% trypsin (Gibco, Germany) for 20 minutes at 37°C. The trypsin was aspirated, and RPE culture medium was added and gently stirred with a pipette to release RPE cells into the medium. RPE cells were collected by centrifugation at 1500 r/min for 4 minutes, resuspended and maintained at 37°C under 5% CO_2_. The hRPE cells were purified using the differential adhesion method, and the degree of purification was determined by RPE65 and ZO-1 (Tight junction protein-1) staining. The human retinal pigment epithelial cell line, ARPE-19, was purchased from the American Type Culture Collection (Manassas, VA, USA).

**Table 1.** Background of Human Doners

Number	Age	Sex	Eye Diseases	Systemic Diseases	Genotype
1	50	Male	N/A	N/A	High risk
2	41	Male	N/A	N/A	Wild-type
3	46	Male	N/A	N/A	High risk
4	38	Male	N/A	N/A	High risk
5	52	Male	N/A	N/A	Wild-type
6	45	Male	N/A	N/A	High risk

N/A, not applicable.

### Sanger Sequencing and Experimental Cell Line Screening

**Figure 1.**
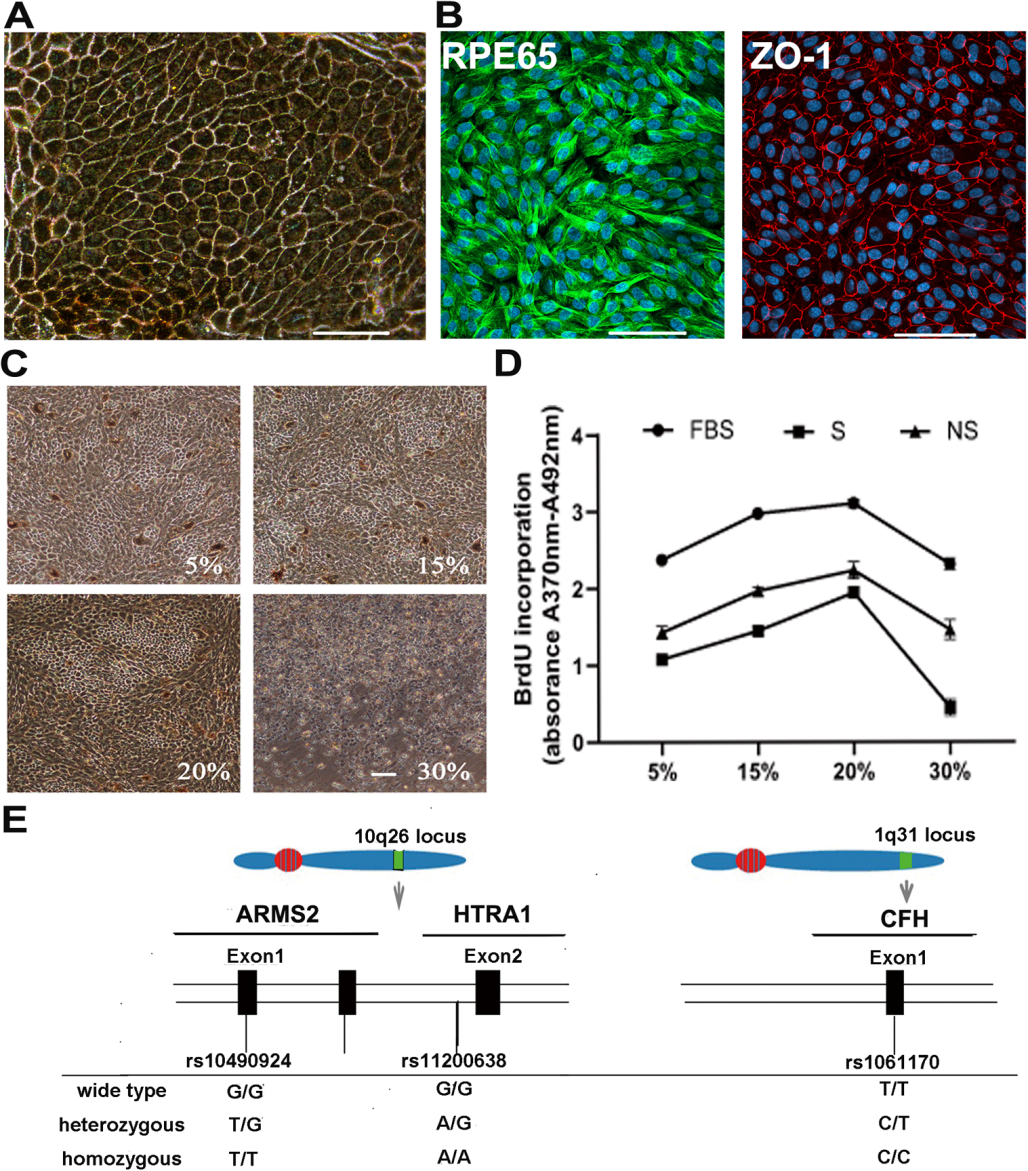
Obtaining allele-specific hRPE cells and determination of the optimum experimental condition. (**A**) The unified hRPE cells appeared as regular hexagonal cells containing pigment under an inverted microscope. Tight junctions were observed among the hexagonal-shaped cells. Scale bars = 100 µm. (**B**) RPE65 (*green*) and ZO-1 (*red*) staining demonstrated that the hexagonal cells were highly purified. Scale bars = 100 µm. (**C, D**) Determination of the optimum action concentration of smokers’ serum. Smokers’ serum was assessed with a concentration gradient of 5%, 15%, 20%, and 30% for 7 days in this experiment. (**C**) The morphological changes of RPE cells under an inverted microscope after exposure to smokers’ serum When the cells were exposed to lower serum concentrations, a stable cell growth status was observed, but many cells detached and floated when the serum concentration increased to 30%. Scale bars = 100 µm. (**D**) The changes in the proliferation of RPE cells after treatment with smokers’ serum were determined by BrdU assays. The results showed that the 20% serum concentration was a turning point. When the serum concentration was higher than 20%, the proliferation of RPE cells starting to decrease. (**E**) Variants at the 10q26 and 1q31 loci, which are associated with the risk of developing AMD. The SNPs rs10490924 in ARMS2 (OMIM #611313) and rs11200638 in HTRA1 (OMIM #602194) are located on chromosome 10q26. The rs1061170 SNP in CFH (OMIM 134370) is located on chromosome 1q31.

Genetic variants in ARMS2 (rs10490924 SNP) and HTRA1 (rs11200638 SNP) have been identified to be strongly associated with the risk of developing AMD (Fig. 1E). Sequence variations were initially identified in donors by next-generation sequencing and subsequently confirmed using Sanger sequencing. Homozygous complement factor H (CFH; rs1061170 SNP) and nonsmoking autopsy eye donors were necessary for this study. Four high-risk cell lines and two low-risk cell lines were obtained based on sequencing results for this study. One pair each of the low-risk (age 41 years) and high-risk RPE cells (age 38 years) from these donors were treated with serum from smokers and nonsmokers in the iTRAQ-based liquid chromatography mass spectrometry pilot study. Then, the selected differentially expressed proteins (DEPs) from the iTRAQ study results were verified with all cell lines.

### Serum Sample Collection and Processing

Sera were obtained from 32 cigarette smokers and 35 healthy nonsmokers. A routine blood examination was conducted, and subjects with blood abnormalities were excluded. Smokers’ sera and nonsmokers’ sera were collected in accordance with the requirements described in Table 2. To eliminate individual differences in the sera, the sera obtained from all smokers and all nonsmokers were pooled separately. Sera were obtained from 32 cigarette smokers and 35 healthy nonsmokers. A routine blood examination was conducted, and the donors with blood abnormalities were excluded. Smokers’ sera and nonsmokers’ sera were collected in accordance with the requirements described in Table 2.

**Table 2.** The Collection Requirements for the Smokers’ Sera and Nonsmokers’ Sera

	Smokers	Nonsmokers
Age	40–60	40–60
Gender	Male	Male
BMI	25–35	25–35
Smoking history, y	>10	0
Cigarettes, per/day	>10	0
The number of people	32	35

BMI, body mass index.

### Optimum Experimental Condition Exposure by BromodeoxyUridine 

To find the optimum experimental conditions for smokers’ serum, a bromodeoxyUridine (BrdU) assay was performed to evaluate cell viability and proliferation ability after serum exposure. Equivalent numbers of RPE cells were cultured in flat-bottomed 96-well plates until they reached 70% confluence. RPE cells were treated with different concentrations of serum (5%, 15%, 20%, and 30%) and proliferation was measured using the BrdU incorporation assay according to the manufacturers’ protocol. Briefly, RPE cells were seeded in a 96-well plate, cultured until they reached 70% confluence. Then, the cells were treated with different concentrations of serum incubate for 72 hours. After the end of the incubation, BrdU was added at a concentration of 10 µM and incorporation of BrdU was quantified by enzyme-linked immunosorbent assay (ELISA) with the BrdU cell proliferation ELISA kit (Roche, Switzerland) according to the manufacturers’ protocol.

### Liquid Chromatography Tandem Mass Spectrometry Sample Preparation for an iTRAQ-Label Experiment

Allele-specific RPE cells obtained from low-risk allele donors and high-risk allele donors were exposed to conditioned medium containing 20% smokers’ serum or nonsmokers’ serum for 7 days and then were processed using iTRAQ-based liquid chromatography tandem mass spectrometry (LC-MS/MS). During the experiment, 1 × 10^5^ cells were planted in each well, and the medium was changed every other day. The time point of day 7 was selected based on the results of the pre-experiment. In the preliminary experiment, we selected the four time points of 3 days, 5 days, 7 days, and 9 days, and observed the cell growth state under the microscope. It was found that the cell growth state was good under the action of the first three time points, whereas a large number of cells appeared apoptosis and vacuolated changes on 9 days. Therefore, we chose the time point when serum acted on RPE cells for the longest time without causing apoptosis, that is, 7 days. Biological replicates were prepared, and the average was used for analysis (Table 3). After the cell pellets were lysed, the total proteins were collected and quantified with a bicinchoninic acid (BCA) assay kit. In brief, each protein sample was reconstituted in dissolution buffer, denatured, reduced, alkylated, and then digested. As previously described,^7^ a variation of the FASP protocol^8^ was used in preparing in-solution digests for proteome analysis.

### iTRAQ Labeling and Strong Cation-Exchange Chromatography

The iTRAQ reagents, which can differentiate cells from each of the experimental conditions, were then added to the solutions in the following manner: iTRAQ reagent 114 to sample LN1, 113 to LN2, 116 to LS1, 115 to LS2, 118 to HN1, 117 to HN2, 121 to HS1, and 119 to HS2. The iTRAQ-labeled peptide samples were fractionated by strong cation-exchange (SCX) chromatography and then collected and subjected to LC-MS/MS analysis.

### LC-MS/MS Analysis Based on Q-Exactive

A desalted peptide mixture was loaded onto an Acclaim Pepmap C18-reversed-phase column (75 µm × 2 cm, 3 µm, 100 Ǻ; Thermo Scientific, Waltham, MA, USA) and separated with a reversed-phase C18 column (75 µm × 10 cm, 5 µm, 300 Ǻ; Agela Technologies) mounted onto a Dionex ultimate 3000 nano LC system. Peptides were eluted using a gradient of 5% to 80% (v/v) acetonitrile in 0.1% formic acid over 45 minutes at a flow rate of 300 nl/min combined with a Q-Exactive mass spectrometer (Thermo Fisher Scientific). The eluates were directly delivered into a Q-Exactive MS in positive ion mode and in a data-dependent manner. To evaluate the performance of this mass spectrometry on iTRAQ-labeled samples, two MS/MS acquisition modes and higher collision energy dissociation (HCD) were used.

### Sequence Database Searching and Bioinformatics Analysis

Thermo Fisher Proteome Discoverer software 1.3/1.4 was applied to process the raw data files and perform database searches. Protein identification was performed against the UniProt_2016_human database using the Mascot search engine (version 2.3.01). A threshold of confidence >95% and a local false discovery rate (FDR) <1% were set for protein identification. A *P* value < 0.05 was required for relative quantification. A functional analysis of Gene Ontology (GO) annotations was performed using the Database for Annotation, Visualization and Integrated Discovery (DAVID) online tool (version 6.7), for the categories of biological process (BP), molecular function (MF), and cellular component (CC). Annotations were considered significant with an FDR-adjusted *P* value < 0.05. Furthermore, these identified proteins were classified and grouped using the Kyoto Encyclopedia of Genes and Genomes (KEGG) database for pathway enrichment analysis.

### Reverse Transcription-Quantitative Polymerase Chain Reaction Analysis

Quantitative real-time polymerase chain reaction (RT-PCR) analyses were performed as previously described.^9^ Briefly, RT-PCR analyses were performed with specific primers in triplicate using a 7900HT Fast Real-Time PCR System-Refurb (Applied Biosystems, Foster City, CA, USA). The cycle number was used to obtain quantitative values and determine the fold difference between different samples. To normalize the genes of interest, glyceraldehyde 3-phosphate dehydrogenase (GAPDH) was simultaneously processed as an internal control. The primer sequences are shown in Table 4. Independent *t*-test analyses were used to compare fold difference values, with significance accepted at *P* < 0.05.

**Table 3.** The Labeling of Samples for the iTRAQ-Based LC-MS/MS Analysis

Serum hRPE	Smoker's Serum (S)	Non-Smoker's Serum (N)
High risk (H)	Sample HS1, HS2	Sample HN1, HN2
Low risk (L)	Sample LS1, LS2	Sample LN1, LN2

* 1 and 2 are biological replicates.

**Table 4.** RT-PCR Primers Sequences

Gene Name	Forward Primer	Reverse Primer
HTRA1, human	5′-TCCCAACAGTTTGCGCCATAA-3′	5′-CCGGCACCTCTCGTTTAGAAA-3′
Caveolin-1, human	5′-GCGACCCTAAACACCTCAAC-3′	5′-ATGCCGTCAAAACTGTGTGTC-3′
GAPDH, human	5′-GAAGGTGAAGGTCGGAGTCA-3′	5′-AATGAAGGGGTCATTGATGG-3′

### Western Blot Analysis

Protein extracts were quantified with a BCA assay kit. Thirty micrograms of each sample were separated on a 10% SDS-PAGE gel and transferred to a PVDF membrane (Millipore, Burlington, MA, USA). The primary antibodies used were anti-caveolin-1 (ab192869, 1:10000; Abcam, UK), anti-HTRA1 (ab199529, 1:100; Abcam, UK), and anti-rhodopsin (ab3267, 1:6000; Abcam, UK). β-actin (TA-09, 1:1000; ZSGB-BIO, China) was used as a loading control. Protein bands were visualized using a conventional enhanced chemiluminescence (ECL) system (GE Healthcare, USA) and quantified with Quantity One (Bio-Rad, USA).

### Immunofluorescence

After specific treatments, the cells were fixed with 4% paraformaldehyde and blocked with 10% normal goat serum in PBS. The primary antibodies used were anti-RPE65 (ab78036, 1:200; Abcam, UK), anti-ZO-1 (21773-1-AP, 1:200; Proteintech, USA) and anti-caveolin-1 (ab192869, 1:500; Abcam, UK). Additionally, 4,6-diamidino-2-phenylindole (DAPI) was used to visualize the cell nuclei. A fluorescence microscope (U-LH100L-3; Olympus Corporation, Tokyo, Japan) was used to examine and capture images.

### Construction of Lentivirus Vector Containing HTRA1 and its Expression in ARPE-19 Cells

A lentivirus vector containing HTRA1 was designed and constructed, and a lentivirus vector without HTRA1 served as a negative control. We transduced ARPE-19 cells with lentivirus carrying HTRA1 with green fluorescence protein (GFP) and obtained stably expressing cells by puromycin selection. The infection rate was determined by the green fluorescence generated by GFP.

### Phagocytosis Assay

Photoreceptor outer segments (POSs) were isolated from fresh bovine eyes and labeled with fluorescein isothiocyanate (FITC), as described earlier.^10^^,^^11^ The RPE cells were co-cultured in conditioned medium containing 200 µg/mL FITC-labeled POSs for 4 hours at 37°C after exposure to the smokers’ serum for 7 days. Then, the excess FITC-labeled POSs were removed by rinsing with PBS, and cells were fixed with paraformaldehyde. The cells were observed and imaged using a fluorescence microscope (U-LH100L-3; Olympus Corporation, Tokyo, Japan). Phagocytosis was quantified by green fluorescence (representing the total POS phagocytosed by RPE cells) with Image J software. Four visual fields from four quadrants were randomly chosen from each group for evaluation. Total proteins were extracted from the RPE cells, which had been cultured in media containing POS, and then rhodopsin was detected by Western blot.

### Data Analysis

Differences between groups were statistically analyzed with independent samples *t* tests. A *P* value < 0.05 was accepted as statistically significant.

## Results

### Obtaining hRPE Cells

After 3 weeks of culture, the cultured autopsied cells exhibited regular hexagonal morphologies with pigment under an inverted microscope (see Fig. 1A). Immunofluorescence staining of ZO-1 and RPE65, an RPE-specific marker, demonstrated that the cells obtained were highly purified human RPE cells (see Fig. 1B).

### The Optimum Experimental Condition is 20% Smokers’ Serum

To maximize the effect of smokers’ serum on RPE cells while avoiding lethal treatment, a concentration gradient was used to screen the optimum smokers’ serum concentration (Fig. 1). We chose 5%, 15%, 20%, and 30% serum concentrations in the cell culture medium. The morphological changes and growth status of RPE cells were observed under an inverted microscope after exposure to smokers’ serum for 7 days. The cell growth status was stable under the lower serum concentrations, but many cells detached and floated when the serum concentration was increased to 30% (see Fig. 1C). The proliferation ability of the RPE cells under different serum concentrations was examined with BrdU assays. The resulting line chart showed that the proliferation of RPE cells was inhibited when the serum concentration exceeded 20% (see Fig. 1D).

### iTRAQ-based LC-MS/MS Analysis Results

**Figure 2.**
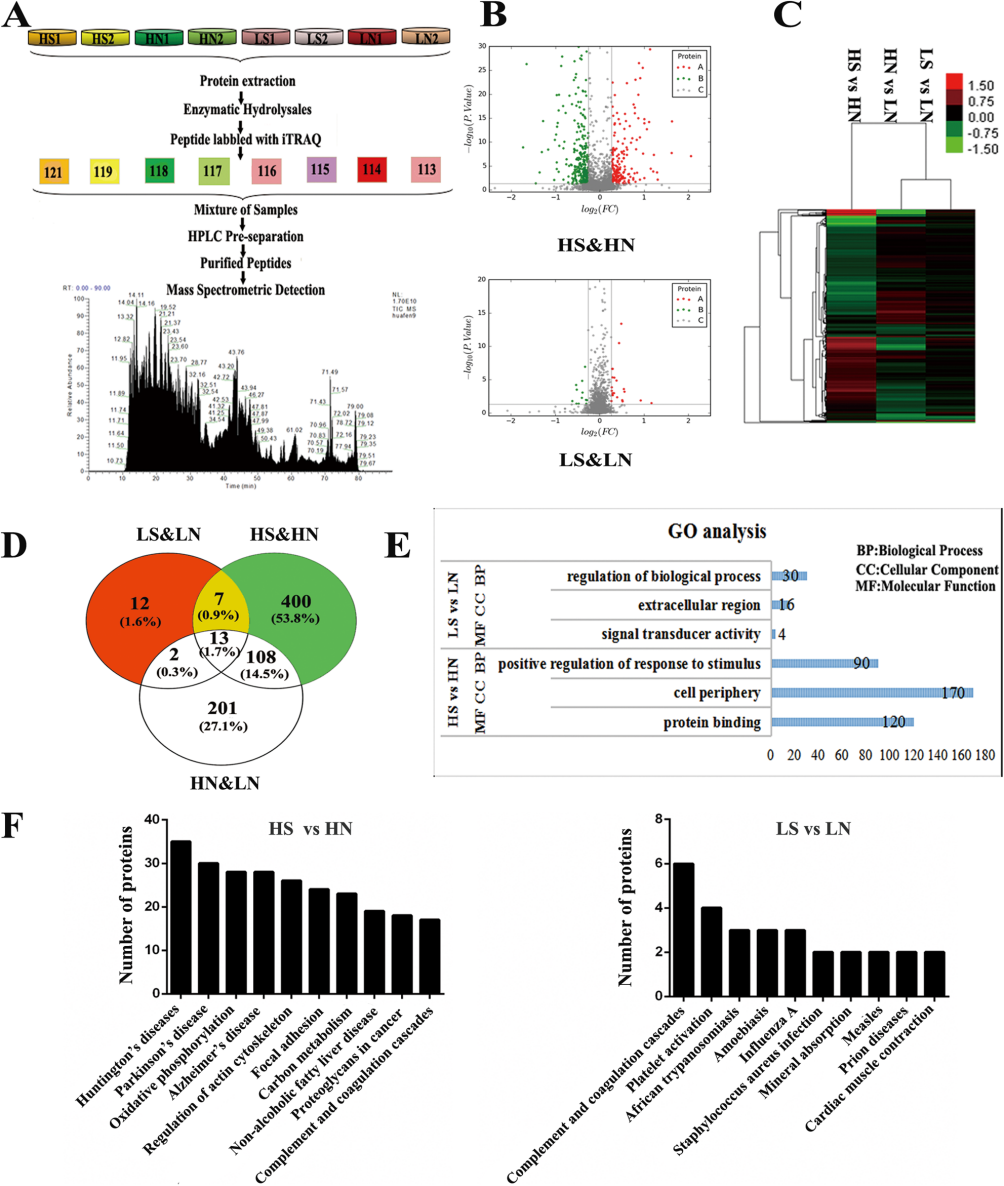
Through proteomic analysis, the DEPs between groups were determined and functional analysis in the high-risk allele (HS versus HN) and low-risk allele (LS versus LN) groups were performed after exposure to smokers’ serum. (**A**) Schematic flowchart of the iTRAQ mass spectrometry method. (**B**) Volcano plot of DEPs. The X-axis represents log2-fold change of high- (positive values) and low-expressed (negative values) proteins. Significant upregulated proteins (log2 [FC] > log2 [1.2] and *P* < 0.05) are represented with red spots, whereas the downregulated proteins (log2 [FC] < log2 [1/1.2] and *P* < 0.05) are represented with green spots. (**C**) Cluster analysis of DEPs in HS versus HN, LS versus LN, and HN versus LN. The protein expression levels are shown as colored boxes, where red indicates an increased expression level, and green indicates a decreased expression level. (**D**) Venn-Euler diagrams of the same DEPs in the high-risk allele group and low-risk allele group after exposure to smokers’ serum. (**E**) GO-enriched analysis for DEPs categorized by biological process (BP), molecular function (MF), and cellular component (CC). The enrichment results with most DEPs gathered and smallest significance level of Fisher's exact test in the BP, MF, and CC are listed in the form. (**F**) Global view of the KEGG enrichment pathways.

Data are available via ProteomeXchange with the identifier PXD009309 (Fig. 2). In this study, 2774 proteins were quantified with 2 or more peptides from the following samples and corresponding iTRAQ reagents: iTRAQ reagent 113 for sample LN2, 114 for LN1, 115 for LS2, 116 for LS1, 117 for HN2, 118 for HN1, 119 for HS2, and 121 for HS1 (Fig. 2A). A total of 464 (HS versus HN) and 30 (LS versus LN) DEPs were identified (ratio < 0.8 or ratio > 1.2, *P* < 0.05; Additional file 1: Supplementary Tables S1, S2). Volcano plots of DEPs after exposure to smokers’ serum were created. Under the effect of smokers’ serum, there were 192 increased proteins and 272 decreased proteins in the high-risk group (HS versus HN). In the low-risk group (LS versus LN), the numbers of increased proteins and decreased proteins were 21 and 9, respectively (see Fig. 2B). A cluster analysis of the DEPs in HS versus HN, LS versus LN, and HN versus LN comparisons is shown in Figure 2C. There were 400 net DEPs in the high-risk group and 12 DEPs in the low-risk group after excluding individual difference-related DEPs (HN versus LN, 324) and synchronously changed proteins (7). The number of significantly changed net DEPs in the high-risk group was much higher than that in the low-risk group (see Fig. 2D). The significant synchronously changed proteins in the high-risk group and low-risk group (ratio < 0.7 or ratio > 1.3 and *P* < 0.05) included seven proteins, as described in Table 5. The caveolin-1 protein and HTRA1 protein were among the net DEPs and significantly more upregulated in the high-risk group than in the low-risk group after exposure to smokers’ serum (Table 6).

**Table 5.** The Synchronous Changed DEPs in the High-Risk Group and Low-Risk Group (Ratio < 0.7 or Ratio > 1.3, and *P* < 0.05)

		HS Versus HN		LS Versus LN	
Accession	Description	*P* Value	Ratio	*P* Value	Ratio
P01742	Ig heavy chain V-I region EU OS = Homo sapiens PE = 1 SV = 1 - [HV101_HUMAN]	4.26E-15	3.077799947	0.000641665	1.474722337
P02671	Fibrinogen alpha chain OS = Homo sapiens GN = FGA PE = 1 SV = 2 - [FIBA_HUMAN]	1.56E-11	1.655900581	3.13E-11	1.354797577
P02675	Fibrinogen beta chain OS = Homo sapiens GN = FGB PE = 1 SV = 2 - [FIBB_HUMAN]	1.34E-10	1.623849773	0.004339253	1.309669728
P02679	Fibrinogen gamma chain OS = Homo sapiens GN = FGG PE = 1 SV = 3 - [FIBG_HUMAN]	2.99E-09	1.761245553	3.98E-14	1.397077314
P09601	Heme oxygenase 1 OS = Homo sapiens GN = HMOX1 PE = 1 SV = 1 - [HMOX1_HUMAN]	0.002377607	1.324064879	0.01229211	1.419950302
P09914	Interferon-induced protein with tetratricopeptide repeats 1 OS = Homo sapiens GN = IFIT1 PE = 1 SV = 2 - [IFIT1_HUMAN]	0.001453819	1.425661915	0.013631126	1.895221335
P20591	Interferon-induced GTP-binding protein Mx1 OS = Homo sapiens GN = MX1 PE = 1 SV = 4 - [MX1_HUMAN]	3.66E-09	1.530872598	0.031798016	2.24634968

**Table 6.** Caveolin-1 and HTRA1: Significantly Upregulated in the High-Risk Group Versus the Low-Risk Group After the Exposure to Smokers’ Serum

Accession	Description	HS Versus HN (High-Risk Group)	LS Versus LN (Low-Risk Group)
Q03135	Caveolin-1 OS = Homo sapiens GN = CAV1 PE = 1 SV = 4 - [CAV1_HUMAN]	1.468536603	0.984785071
Q92743	Serine protease HTRA1 OS = Homo sapiens GN = HTRA1 PE = 1 SV = 1 - [HTRA1_HUMAN]	1.725795716	0.991192547

A global functional view of DEPs was obtained by a GO functional classification annotation and KEGG metabolic pathway analysis. The GO annotation analysis included BP, MF, and CC categories according to the GO database (Additional file 2: Supplementary Figs. S1A, S1B, S1C). In the high-risk group (HS versus HN), positive regulation of response to stimulus, protein binding, and cell periphery were the most abundant categories in BP, MF, and CC, respectively. In the low-risk group (LS versus LN), the most abundant categories in BP, MF, and CC were regulation of biological process, signal transducer activity, and extracellular region, respectively (see Fig. 2E). To provide further understanding of the DEPs, they were mapped to KEGG pathways based on their KEGG gene IDs. Furthermore, KEGG pathway enrichment analysis revealed that the top six significantly changed pathways in the high-risk group (HS versus HN) were involved in Huntington's disease (35), Parkinson's disease (30), oxidative phosphorylation (28), Alzheimer's disease (AD) (28), regulation of the actin cytoskeleton (26), and focal adhesion (24; Additional file 3: Supplementary Fig. S2). In the low-risk group (LS versus LN), the top two significantly changed pathways were complement and coagulation cascades (6) and platelet activation (4; see Fig. 2F).

### Validation of Caveolin-1 Expression in RPE Cells After Exposure to Smokers’ Serum

**Figure 3.**
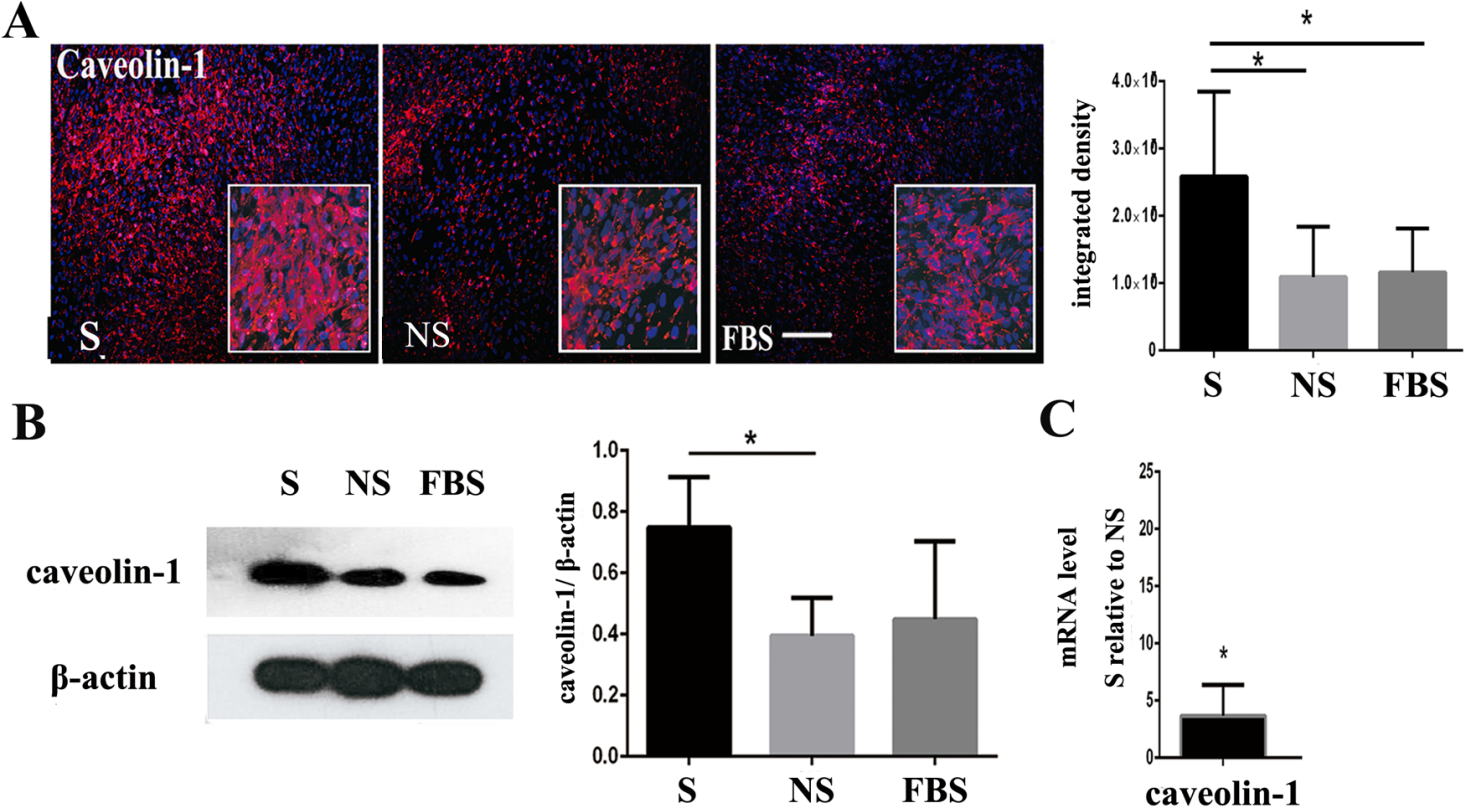
Under the stimulation of smokers’ serum, the expression of caveolin-1 was upregulated. (**A**) Immunofluorescence results of caveolin-1 expression after exposure to smokers’ serum. The positive rate of fluorescence staining and average fluorescence intensity of caveolin-1 in the S group were higher than those in the NS and FBS groups. Scale bars = 200 µm. (**B**) The expression level of caveolin-1 protein in the S group was significantly higher than that in the NS and FBS groups (*P* < 0.05). β-actin was used as a loading control. (**C**) Gene expression changes following smokers’ serum exposure by quantitative RT-PCR. Compared with the NS group, the S group showed significant changes in the mRNA levels of caveolin-1, which was in line with the mass spectrometry results.

The iTRAQ-based proteomics results showed that the caveolin-1 protein and HTRA1 protein were significantly more upregulated in the high-risk group than that in the low-risk group after exposure to smokers’ serum (see Table 5). There may be an interplay between the AMD high-risk alleles and cigarette smoking on the AMD process, we detected the expression of caveolin-1 when RPE cells were exposed to smokers’ serum or overexpressing HTRA1 independently. Quantitative RT-PCR, immunofluorescence staining, and Western blot analyses were used to validate the caveolin-1 expression after exposure of the RPE cells to smokers’ serum (S), nonsmokers’ serum (NS), and fetal bovine serum (FBS). Caveolin-1 staining revealed that the number of positive cells and integrated intensity in the S group were the highest of the three groups (Fig. 3A). As shown in Figure 3B, the expression level of the caveolin-1 protein was significantly higher in the S group than that in the NS and FBS groups (*P* < 0.05). The gene expression changes determined by RT-PCR are shown in Figure 3C. Compared with the NS group, the S group showed significant high level mRNA expression of caveolin-1, which was consistent with the iTRAQ-based proteomics results (Fig. 3).

### Validation of Caveolin-1 Expression in RPE Cells After Overexpressing HTRA1

**Figure 4.**
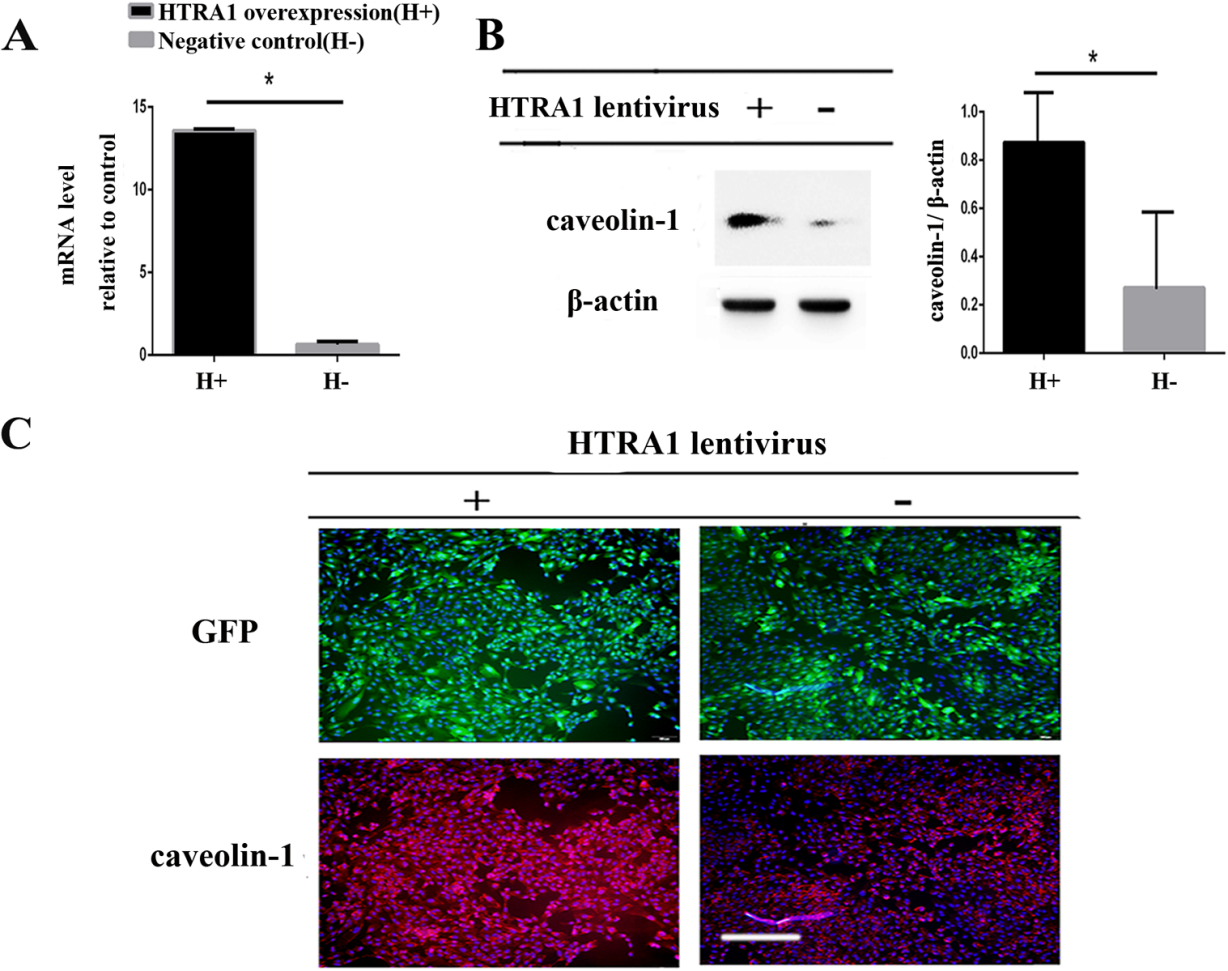
Overexpression of HTRA1 increased caveolin-1 protein expression. (**A**) ARPE-19 cells were transduced with lentivirus containing HTRA1 (H+). A virus without HTRA1 served as the negative control (H-). RT-PCR results demonstrated that the expression of HTRA1, compared to the expression in negative control cells, was significantly up-regulated (up to 13-fold) in the lentivirus-treated cells. (**B**) Caveolin-1 protein expression was detected in the HTRA1-overexpression cells by Western blot. The quantity of caveolin-1 protein in the HTRA1-overexpression group was higher than that in the negative control group. β-actin was used as a loading control. (**C**) Detection of caveolin-1 protein expression by immunofluorescence. Scale bars = 100 µm. The expression of caveolin-1 increased in HTRA1-overexpressing ARPE-19 cells, which was consistent with the Western blot results.

GFP- and HTRA1-overexpressing lentivirus-transduced ARPE-19 cells were obtained by puromycin selection and were used to detect caveolin-1 expression. RT-PCR results demonstrated that, compared to the expression in negative control (H-) cells, the expression of HTRA1 was significantly upregulated (up to 13-fold) in the lentivirus containing HTRA1-treated cells (Fig. 4A). The expression of caveolin-1 protein, related to an AMD high-risk allele, HTRA1, was then verified by Western blot and immunofluorescence. The overexpression of HTRA1 increased caveolin-1 protein expression, as shown in Figures 4B and 4C. The expression of caveolin-1 increased when RPE cells were exposed to smokers’ serum or overexpressing HTRA1 (Fig. 4).

### Smokers’ Serum Enhanced RPE Phagocytic Function

**Figure 5.**
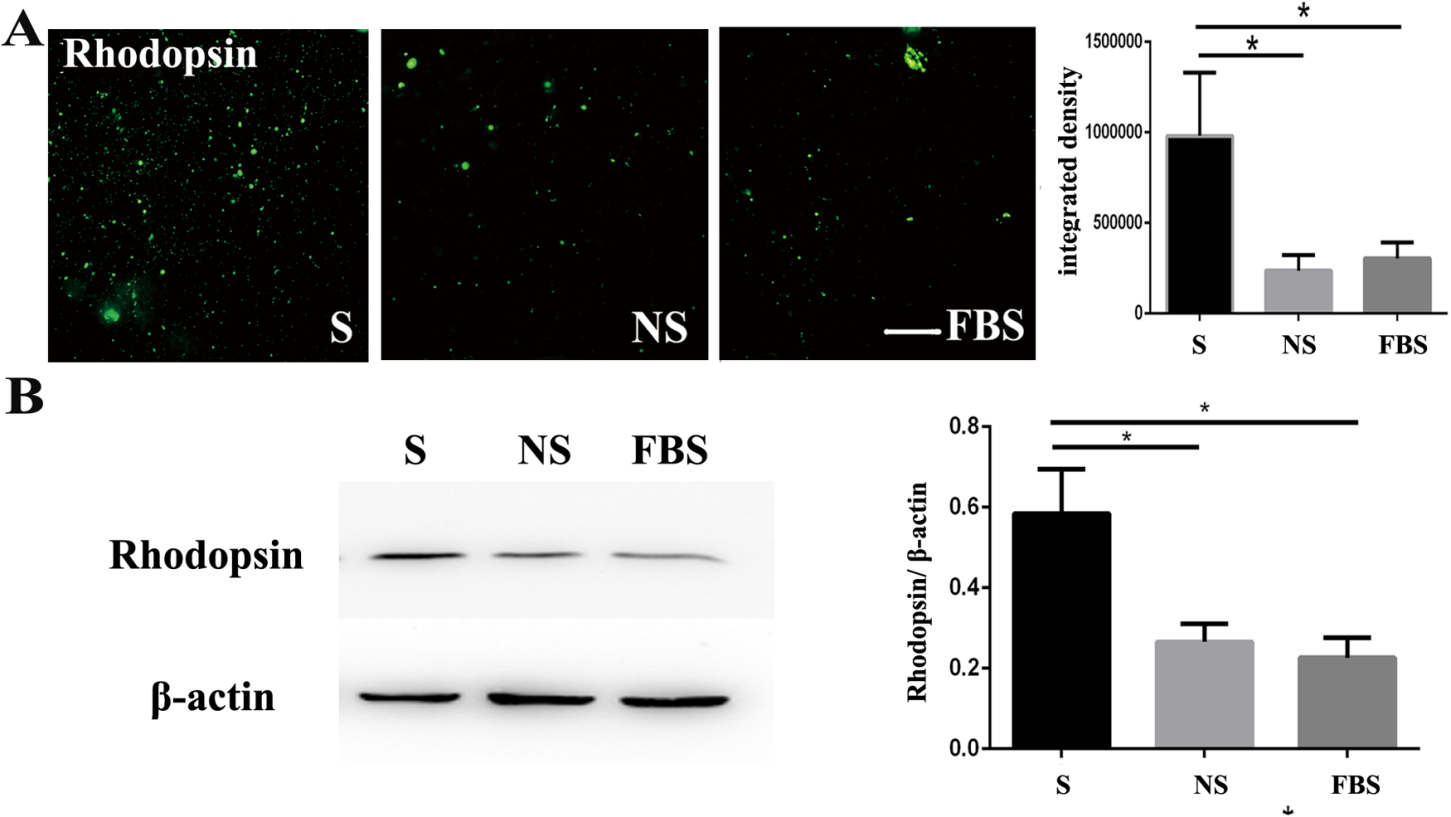
After the stimulation of smokers’ serum, the phagocytosis function of the RPE increased. (**A**) The number of FITC-labeled POS engulfed by ARPE-19 cells was compared by fluorescence microscopy. The number of rhodopsin labelled with FITC in the S group was more than that in the NS and FBS groups. Nuclei were stained by DAPI. Scale bars = 200 µm. (**B**) The rhodopsin protein exhibited higher expression after exposure to smokers’ serum.

Previous studies have shown that caveolin-1 is associated with phagocytosis and phagolysosomal digestion,^12^ the ablation of which may result in the accumulation of bis-retinoid fluorophores, such as A2E and all-trans-retinal dimers, further promoting AMD. Therefore, we estimated the phagocytic function of RPE cells after exposure to smokers’ serum. The number of phagocytes containing POS labeled with FITC in the high-risk RPE cells were observed using a fluorescence microscope. The number of phagocytes containing POS (rhodopsin) in the smokers’ serum (S) group was higher than that in the nonsmokers’ serum (NS) and fetal bovine serum (FBS) groups (Fig. 5A). Western blots also showed that rhodopsin protein expression increased after exposure to smokers’ serum (see Fig. 5B). Overall, the phagocytosis of RPE cells was enhanced after the stimulation of smokers’ serum (Fig. 5).

## Discussion

AMD is a common macular disease affecting elderly people, which ranks third as a cause of blindness after cataract and glaucoma.^13^ At present, there is no effective treatment methods. The biggest obstacle to developing treatments is the lack of understanding of the molecular basis of disease. In our study, allele-specific RPE cells directly exposed to culture medium containing smokers’ serum were used as an AMD disease model for comparison. Specific, genotyped, human autopsy RPE cells were used in this study. In vivo mouse model studies may be another effective way to assess cigarette smoke damage in the retina.^14^ However, some genes are expressed only in primates, such as the ARMS2 gene, and the lack of an anatomic macula in mice limits the use of mouse models for studies of AMD.^15^ Particulate or aqueous cigarette smoke extracts are commonly used as exposure agents to study the in vitro effects of cigarette smoking on RPE cells.^16^^,^^17^ Because cigarette smoke contains numerous potential oxidants, including nitric oxide, carbon monoxide, and many other toxic chemical moieties, complicated chemical reactions occur in the serum. Extracts alone may not be an ideal high-risk environmental factor exposure. In this study, we collected serum mixtures from 32 prescreened smokers as the exposure agent to achieve maximum physical characteristics and minimum individual differences. The optimal dosage of the smokers’ serum should have the most toxic effect but avoid cell death. In our study, we concluded that a serum concentration of 20% was best.

An iTRAQ-based proteomics analysis showed that the proteomic changes in the high-risk group were greater than those in the low-risk group with smokers’ serum stimulation. According to the KEGG pathway enrichment analysis, the significant pathway changes in the high-risk group involved Alzheimer's disease, oxidative phosphorylation, regulation of actin cytoskeleton, and focal adhesion. As the retina is an integral part of the central nervous system (CNS), previous clinical studies have found numerous links between Alzheimer's disease and AMD.^18^ Notably, one study showed that heterogeneity may be related to the effect of smoking on the risk of developing Alzheimer's disease,^19^ suggesting possible links between the high-risk alleles and cigarette smoking in the occurrence and development of AMD. The changes in oxidative phosphorylation, actin cytoskeleton, and focal adhesion were involved in cigarette smoke-induced oxidative stress, consistent with a previous study.^20^ However, this stress effect was more obvious in the high-risk group in our study. In the low-risk group, the top two significant pathways were complement and coagulation cascades and platelet activation, which are largely involved in complement system reactions (see Fig. 2F). Complement is an important component of the innate immune system, which may have a protective effect on the RPE cells exposed to smokers’ serum. Kunchithapautham's study came to the conclusion that smoke exposure causes oxidative stress and complement to act synergistically in the pathogenesis of AMD, which is in line with our results.^17^

Our study showed there are interactions between AMD high-risk alleles, HTRA1, and cigarette smoking in promoting AMD progress by upregulating caveolin-1 expression. HTRA1 is a serum protease that may modulate AMD pathogenesis through multiple pathways. The previous study shows that HTRA1 plays an important role in choroidal neovascularization for patients with AMD. Vascular endothelial growth factor (VEGF) were downregulated in HTRA1 knockout (HTRA1-/-) mice. Overexpression of HTRA1 could change the integrity of Bruch's membrane and accelerate the development of CNV. In addition, HTRA1 expression seems to be coupled to oxidative stress and inflammation in the retina. In addition, when HTRA1 is overexpressed in mice, the elastic network of extracellular matrix in Bruch's membrane becomes fragmented between the RPE and the choroid. In our study, we found that HTRAI combined with smoking can upregulate the expression of caveolin-1. Several studies indicate that caveolin-1 belongs to the group of senescence-associated genes, which play a central role in promoting cellular senescence. Overexpressing caveolin-1 repressed the proliferation of cells and led to a senescence-associated increase in beta-galactosidase activity.^21^ In addition, another previous study showed that downregulation of caveolin-1 may be important for the RPE cells to prevent cellular stress-induced apoptotic cell death, a condition implicated in the early pathogenesis of AMD.^22^ Some research has been done previously on the effect of genetic and environmental factors in the pathogenesis of AMD. An epidemiological study concluded that cigarette smoking is related to the long-term incidence and progression of AMD.^3^ Additionally, a significant association between single-nucleotide polymorphisms (SNPs) in the ARMS2 and HTRA1 genes and the risk of AMD was previously identified by some studies.^4^^,^^5^ Regarding the relationship between genetic factors and cigarette smoking, some researchers believe that cigarette smoking is an independent risk factor for AMD and not associated with genetic factors.^2^ Chakravarthy's study found that with smoking under the same conditions, the incidence in patients with advanced AMD with the ARMS2 high-risk allele was 10 times higher than that of patients with the wild-type allele, whereas CFH was associated only with the occurrence of early AMD.^23^ In addition, Nakayama's study found that HTRA1 overexpression in cigarette smoke-exposed mice led to CNV, which is an important characteristics of wet AMD.^6^ Whereas our research confirmed that there are synergic interactions between AMD high-risk alleles and cigarette smoking in their effect on the expression of caveolin-1, and these interactions may promote the occurrence and development of AMD.

In addition, we found that the phagocytosis function of RPE cells was enhanced after exposure to smokers’ serum, which may suggest a self-defense mechanism in response to external insult and oxidate stress. In several previous studies, inflammation has been thought to be associated with the pathogenesis of AMD, which results from an impairment of intracellular cleansing systems. The accumulation or elimination of waste material or the subsequent inflammatory reaction eventually leads to the destruction of light-sensing cells.^24^ Similarly, there are studies that showed that the phagocytic function of the retinal pigment epithelium provides an additional oxidative burden in AMD. Accumulation of lipofuscin is one of the most characteristic features in aged RPE cells, involved in the pathogenesis of AMD.^25^^,^^26^ In summary, we confirmed that the AMD high-risk alleles and AMD high-risk environmental factor, namely cigarette smoking, can promote the occurrence and development of AMD by regulating caveolin-1 expression, upregulation of which will induce apoptotic cell death in response to cellular stress in early AMD conditions. In addition, the enhanced phagocytosis function after exposure to smokers’ serum may be related to the AMD pathologic process in cigarette smokers.

## Supplementary Material

Supplement 1

Supplement 2

Supplement 3

## References

[1] Gheorghe A, Mahdi L, Musat O. Age-Related Macular Degeneration. *Rom J Ophthalmol*. 2015; 59: 74–77. 26978865 PMC5712933

[2] Velilla S, Garcíalayana A, Dolzmarco R, et al. Smoking and Age-Related Macular Degeneration: Review and Update. *J Ophthalmol*. 2013; 2013: 895147. 24368940 10.1155/2013/895147 PMC3866712

[3] Klein R, Knudtson MD, Cruickshanks KJ, Klein BE. Further observations on the association between smoking and the long-term incidence and progression of age-related macular degeneration: the Beaver Dam Eye Study. *Arch Ophthalmol*. 2008; 126: 115. 18195228 10.1001/archopht.126.1.115

[4] Jian T, Yu W , Qin X, et al. Association of genetic polymorphisms and age-related macular degeneration in Chinese population. *Invest Ophthalmol Vis Sci*. 2011; 53: 4262–4269. 10.1167/iovs.11-8542 22618592

[5] Abbas RO, Azzazy HM. Association of single nucleotide polymorphisms in CFH, ARMS2 and HTRA1 genes with risk of age-related macular degeneration in Egyptian patients. *Ophthalmic Genetics*. 2013; 34: 209–216. 23362846 10.3109/13816810.2012.762934

[6] Nakayama M, Iejima D, Akahori M, Kamei J, Goto A, Iwata T. Overexpression of HtrA1 and exposure to mainstream cigarette smoke leads to choroidal neovascularization and subretinal deposits in aged mice. *Invest Ophthalmol Vis Sci*. 2014; 55: 6514. 25205867 10.1167/iovs.14-14453

[7] Bileck A, Kreutz D, Muqaku B, Slany A, Gerner C. Comprehensive assessment of proteins regulated by dexamethasone reveals novel effects in primary human peripheral blood mononuclear cells. *J Proteome Res*. 2014; 13: 5989–6000. 25347463 10.1021/pr5008625

[8] Wiśniewski JR, Zougman A, Nagaraj N, Mann M. Universal sample preparation method for proteome analysis. *Nat Methods*. 2009; 6: 359–362. 19377485 10.1038/nmeth.1322

[9] Lohr HR, Kuntchithapautham K, Sharma AK, Rohrer B. Multiple, parallel cellular suicide mechanisms participate in photoreceptor cell death. *Exp Eye Res*. 2006; 83: 380–389. 16626700 10.1016/j.exer.2006.01.014

[10] Molday RS, Hicks D, Molday L. Peripherin. A rim-specific membrane protein of rod outer segment discs. *Invest Ophthalmol Vis Sci*. 1987; 28: 50. 2433249

[11] Finnermann SC, Bonilha VL, Marmorstein AD, Rodriguez-Boulan E. Phagocytosis of rod outer segments by retinal pigment epithelial cells requires alpha(v)beta5 integrin for binding but not for internalization. *Proc Natl Acad Sci USA*. 1997; 94: 12932–12937. 9371778 10.1073/pnas.94.24.12932 PMC24241

[12] Gu X, Reagan AM, Mcclellan ME, Elliott MH. Caveolins and caveolae in ocular physiology and pathophysiology. *Prog Retin Eye Res*. 2017; 56: 84–106. 27664379 10.1016/j.preteyeres.2016.09.005 PMC5237608

[13] Cai B, Li Z, Sun S, et al. Novel mutations in the OPN1LW and NR2R3 genes in a patient with blue cone monochromacy. *Ophthalmic Genet*. 2019; 40: 43–48 30614359 10.1080/13816810.2018.1561902

[14] Fujihara M, Nagai N, Sussan TE, Biswal S, Handa TJ. Chronic cigarette smoke causes oxidative damage and apoptosis to retinal pigmented epithelial cells in mice. *PLoS One*. 2008; 3: e3119. 18769672 10.1371/journal.pone.0003119 PMC2518621

[15] Marmorstein AD, Marmorstein LY. The challenge of modeling macular degeneration in mice. *Trends in Genetics*. 2007; 23: 225–231. 17368622 10.1016/j.tig.2007.03.001

[16] Yu AL, Burger J , Welge-Lussen U. Biological Effects of Cigarette Smoke in Cultured Human Retinal Pigment Epithelial Cells. *PLoS One*. 2012; 7: e48501. 23155386 10.1371/journal.pone.0048501 PMC3498276

[17] Kunchithapautham K, Atkinson C, Rohrer B. Smoke exposure causes endoplasmic reticulum stress and lipid accumulation in retinal pigment epithelium through oxidative stress and complement activation. *J Biolog Chem*. 2014; 289: 14534. 10.1074/jbc.M114.564674 PMC4031511 24711457

[18] Wong WL, Su X, Li X, et al. Global prevalence of age-related macular degeneration and disease burden projection for 2020 and 2040: a systematic review and meta-analysis. *Lancet Global Health*. 2014; 2: e106. 25104651 10.1016/S2214-109X(13)70145-1

[19] Xu W, Tan L, Wang HF, et al. Meta-analysis of modifiable risk factors for Alzheimer's disease. *J Neurol Neurosurg Psychiatry*. 2015; 86: 1299. 26294005 10.1136/jnnp-2015-310548

[20] Bertram KM, Baglole CJ, Phipps RP, Libby RT. Molecular regulation of cigarette smoke induced-oxidative stress in human retinal pigment epithelial cells: implications for age-related macular degeneration. *Am J Physiol Cell Physiol*. 2009; 297: C1200. 19759330 10.1152/ajpcell.00126.2009 PMC2777395

[21] Volonte D, Zhang K, Lisanti MP, Galbiati F. Expression of caveolin-1 induces premature cellular senescence in primary cultures of murine fibroblasts. *Mol Biol Cell*. 2002; 13: 2502–2517. 12134086 10.1091/mbc.01-11-0529 PMC117330

[22] Kook D, Wolf AH, Yu AL, et al. The protective effect of quercetin against oxidative stress in the human RPE in vitro. *Invest Ophthalmol Vis Sci*. 2008; 49: 1712–1720. 18385095 10.1167/iovs.07-0477

[23] Chakravarthy U, Mckay GJ, de Jong PT, et al. ARMS2 increases the risk of early and late age-related macular degeneration in the European Eye Study. *Ophthalmology*. 2013; 120: 342. 23098369 10.1016/j.ophtha.2012.08.004

[24] Kivinen N, Koskela A, Kauppinen A, Kaarniranta K. Pathogenesis of age-related macular degeneration - dialogue between autophagy and inflammasomes. *Duodecim; laaketieteellinen aikakauskirja*. 2017; 133: 641. 29243450

[25] Ben-Shabat S, Parish CA, Hashimoto M, Liu J, Nakanishi K, Sparrow JR. Fluorescent pigments of the retinal pigment epithelium and age-related macular degeneration. *Bioorg Med Chem Lett*. 2001; 11: 1533–1540. 11412975 10.1016/s0960-894x(01)00314-6

[26] Kennedy CJ, Rakoczy PE, Constable IJ. Lipofuscin of the retinal pigment epithelium: a review. *Eye (Lond)*. 1995; 9(Pt 6): 763–771. 8849547 10.1038/eye.1995.192

