# Supplementary material for: A Pilot Application of an iTRAQ-Based Proteomics Screen Estimates the Effects of Cigarette Smokers’ Serum on RPE Cells With AMD High-Risk Alleles
Source: Transl Vis Sci Technol. 2022 Feb 9;11(2):15. doi: 10.1167/tvst.11.2.15 (PMC8842534; doi:10.1167/tvst.11.2.15)
Supplement: Supplement 1 [file tvst-11-2-15_s001.pdf]

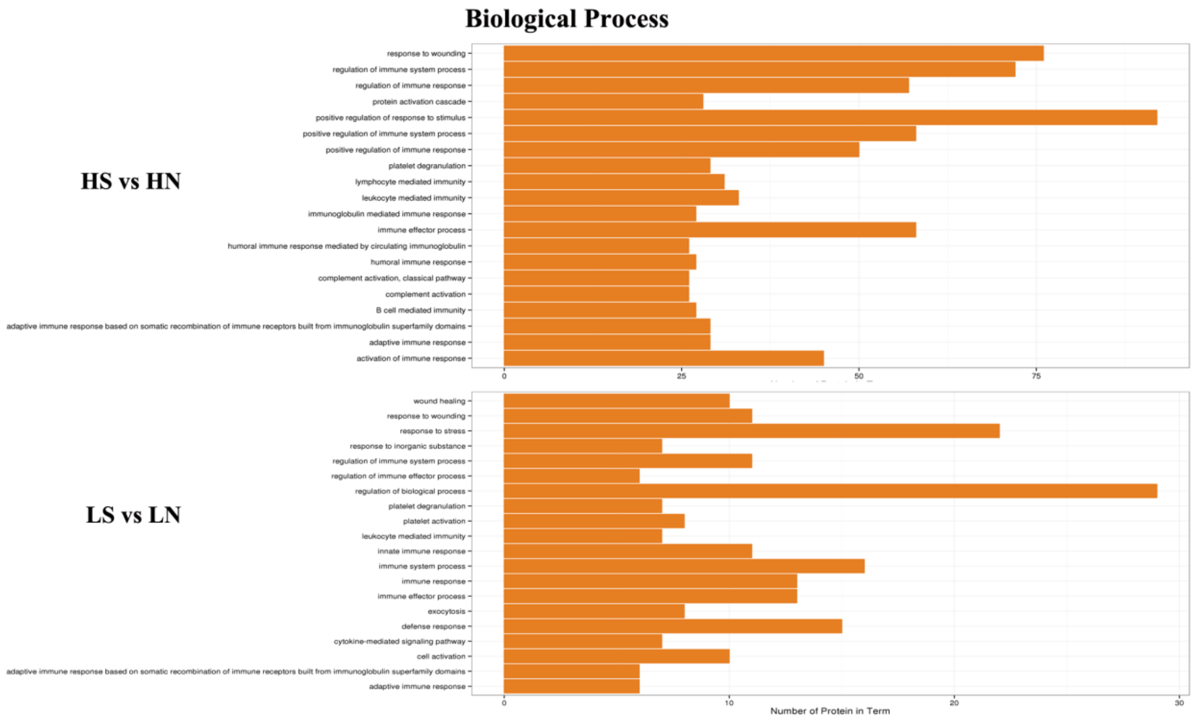
Biological Process
HS vs HN
response to wounding
regulation of immune system process
regulation of immune response
protein activation cascade
positive regulation of response to stimulus
positive regulation of immune system process
positive regulation of immune response
platelet degranulation
lymphocyte mediated immunity
leukocyte mediated immunity
immunoglobulin mediated immune response
immune effector process
humoral immune response mediated by circulating immunoglobulin
humoral immune response
complement activation, classical pathway
complement activation
B cell mediated immunity
adaptive immune response based on somatic recombination of immune receptors built from immunoglobulin superfamily domains
adaptive immune response
activation of immune response
0
25
50
75
LS vs LN
wound healing
response to wounding
response to stress
response to inorganic substance
regulation of immune system process
regulation of immune effector process
regulation of biological process
platelet degranulation
platelet activation
leukocyte mediated immunity
innate immune response
immune system process
immune response
immune effector process
exocytosis
defense response
cytokine-mediated signaling pathway
cell activation
adaptive immune response based on somatic recombination of immune receptors built from immunoglobulin superfamily domains
adaptive immune response
0
10
20
30
Number of Protein in Term

S1a

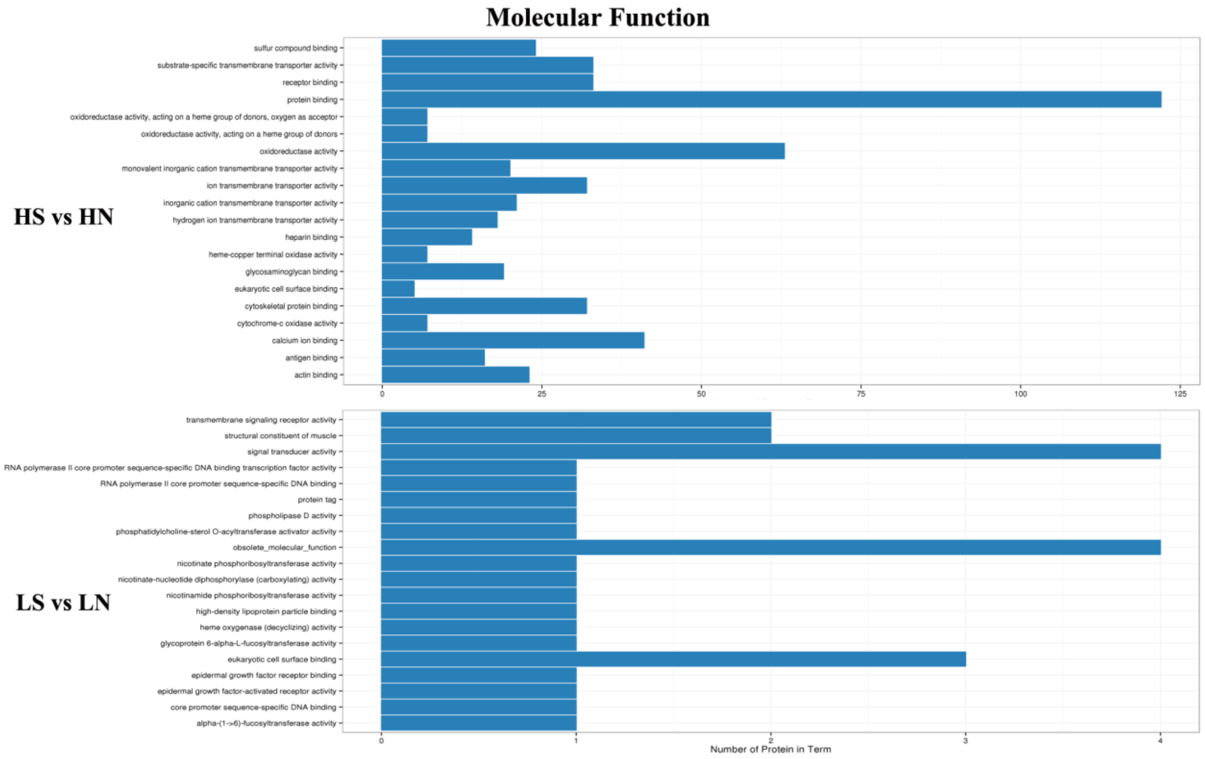
Molecular Function
HS vs HN
sulfur compound binding
substrate-specific transmembrane transporter activity
receptor binding
protein binding
oxidoreductase activity, acting on a heme group of donors, oxygen as acceptor
oxidoreductase activity, acting on a heme group of donors
oxidoreductase activity
monovalent inorganic cation transmembrane transporter activity
ion transmembrane transporter activity
inorganic cation transmembrane transporter activity
hydrogen ion transmembrane transporter activity
heparin binding
heme-copper terminal oxidase activity
glycosaminoglycan binding
eukaryotic cell surface binding
cytoskeletal protein binding
cytochrome-c oxidase activity
calcium ion binding
antigen binding
actin binding
0
25
50
75
100
125
LS vs LN
transmembrane signaling receptor activity
structural constituent of muscle
signal transducer activity
RNA polymerase II core promoter sequence-specific DNA binding transcription factor activity
RNA polymerase II core promoter sequence-specific DNA binding
protein tag
phospholipase D activity
phosphatidylcholine-sterol O-acyltransferase activator activity
obsolete_molecular_function
nicotinate phosphoribosyltransferase activity
nicotinate-nucleotide diphosphorylase (carboxylating) activity
nicotinamide phosphoribosyltransferase activity
high-density lipoprotein particle binding
heme oxygenase (decyclizing) activity
glycoprotein 6-alpha-L-fucosyltransferase activity
eukaryotic cell surface binding
epidermal growth factor receptor binding
epidermal growth factor-activated receptor activity
core promoter sequence-specific DNA binding
alpha-(1->6)-fucosyltransferase activity
0
1
2
3
4
Number of Protein in Term

S1b

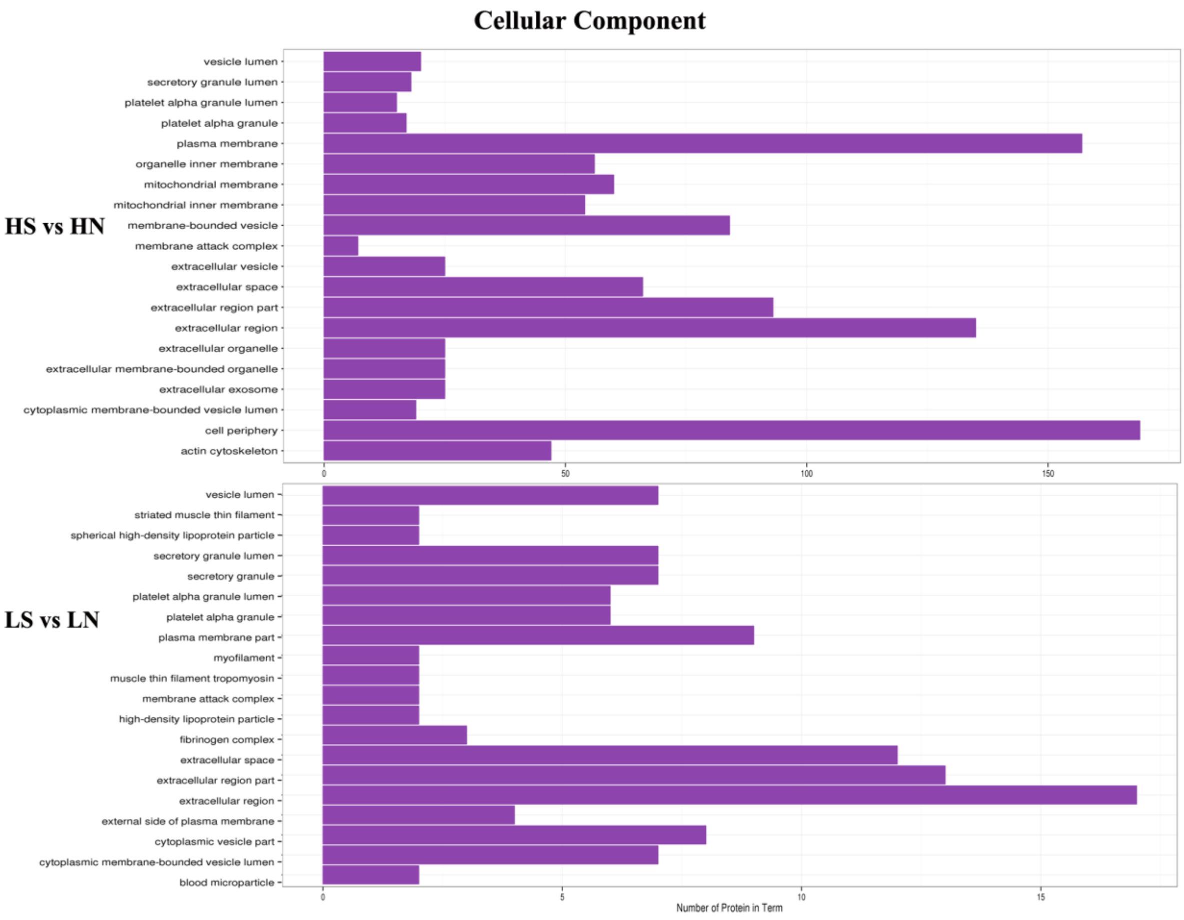

S1c

Supplementary Figure S1. GO annotation of the main altered proteins in the high-risk group and low-risk group: (A) Biological Process; (B) Molecular Function; and (C) Cellular Component.
