# Supplementary figures and images for: A Pilot Application of an iTRAQ-Based Proteomics Screen Estimates the Effects of Cigarette Smokers’ Serum on RPE Cells With AMD High-Risk Alleles
Source: Transl Vis Sci Technol. 2022 Feb 9;11(2):15. doi: 10.1167/tvst.11.2.15 (PMC8842534; doi:10.1167/tvst.11.2.15)

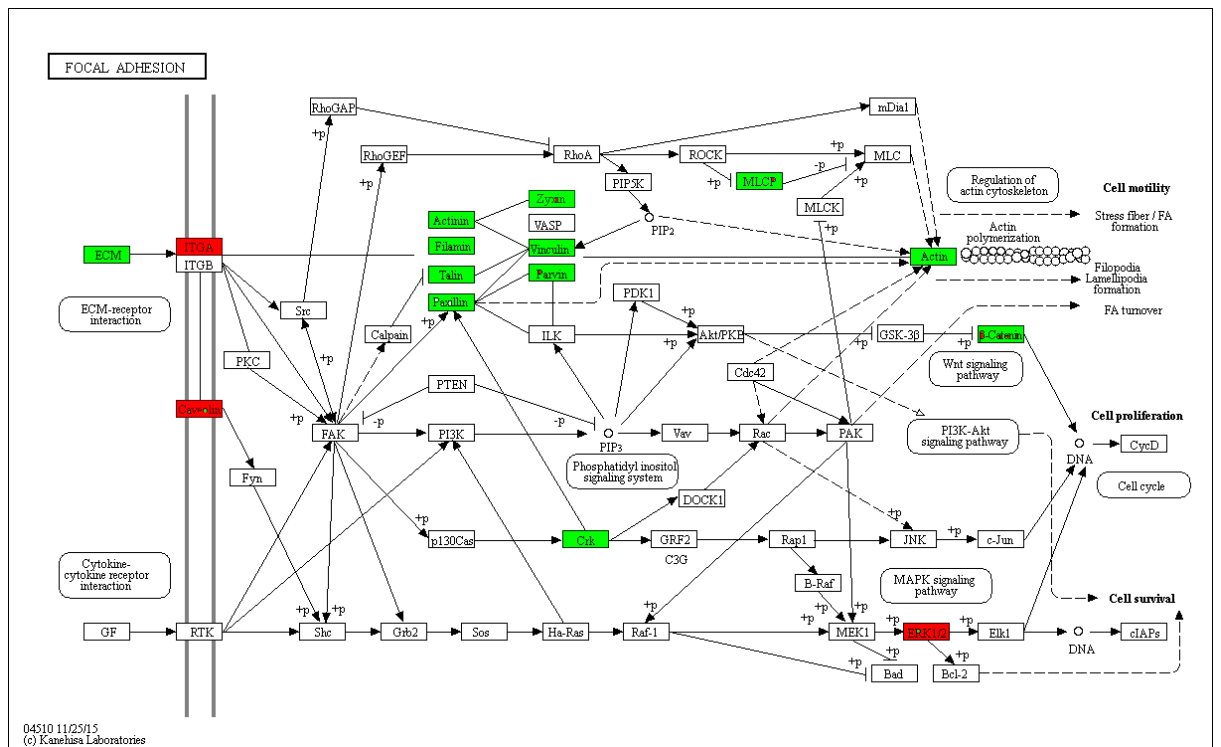

**Supplementary Figure S2.** Focal adhesion pathways.

Supplement: Supplement 2 [file tvst-11-2-15_s002.pdf]
