# Supplementary material for: A Pilot Application of an iTRAQ-Based Proteomics Screen Estimates the Effects of Cigarette Smokers’ Serum on RPE Cells With AMD High-Risk Alleles
Source: Transl Vis Sci Technol. 2022 Feb 9;11(2):15. doi: 10.1167/tvst.11.2.15 (PMC8842534; doi:10.1167/tvst.11.2.15)
Supplement: Supplement 3 [file tvst-11-2-15_s003.pdf]

Table S1 Differentially expressed proteins in high risk group after smoker serum stimulation (HS vs HN)(ratio<0.8 or ratio>1.2, P<0.05)

Accession	Description	p_value	ratio
**Q92743**	**Serine protease HTRA1 OS=Homo sapiens GN=HTRA1 PE=1 SV=1 - [HTRA1_HUMAN]**	**8.92E-17**	**1.725795716**
**Q03135**	**Caveolin-1 OS=Homo sapiens GN=CAV1 PE=1 SV=4 - [CAV1_HUMAN]**	**0.0000126**	**1.468536603**
Q15942	Zyxin OS=Homo sapiens GN=ZYX PE=1 SV=1 - [ZYX_HUMAN]	2.13E-08	0.732883026
Q7Z2W4	Zinc finger CCCH-type antiviral protein 1 OS=Homo sapiens GN=ZC3HAV1 PE=1 SV=3 - [ZCCHV_HUMAN]	0.049020875	1.221001221
Q93050	V-type proton ATPase 116 kDa subunit a isoform 1 OS=Homo sapiens GN=ATP6V0A1 PE=1 SV=3 - [VPP1_HUMAN]	0.0000256	1.229130391
Q9Y277	Voltage-dependent anion-selective channel protein 3 OS=Homo sapiens GN=VDAC3 PE=1 SV=1 - [VDAC3_HUMAN]	0.0000355	1.210130521
P45880	Voltage-dependent anion-selective channel protein 2 OS=Homo sapiens GN=VDAC2 PE=1 SV=2 - [VDAC2_HUMAN]	1.42E-20	1.263489609
P21796	Voltage-dependent anion-selective channel protein 1 OS=Homo sapiens GN=VDAC1 PE=1 SV=2 - [VDAC1_HUMAN]	4.33E-40	1.302607901
P04004	Vitronectin OS=Homo sapiens GN=VTN PE=1 SV=1 - [VTNC_HUMAN]	2.76E-45	2.352394109
P18206	Vinculin OS=Homo sapiens GN=VCL PE=1 SV=4 - [VINC_HUMAN]	2.94E-74	0.65593577
P08670	Vimentin OS=Homo sapiens GN=VIM PE=1 SV=4 - [VIME_HUMAN]	0	0.431886289
P13611	Versican core protein OS=Homo sapiens GN=VCAN PE=1 SV=3 - [CSPG2_HUMAN]	5.23E-18	0.725382991
Q9Y224	UPF0568 protein C14orf166 OS=Homo sapiens GN=C14orf166 PE=1 SV=1 - [CN166_HUMAN]	0.003431244	0.64821417
Q14376	UDP-glucose 4-epimerase OS=Homo sapiens GN=GALE PE=1 SV=2 - [GALE_HUMAN]	0.0000108	1.294498382
Q13404	Ubiquitin-conjugating enzyme E2 variant 1 OS=Homo sapiens GN=UBE2V1 PE=1 SV=2 - [UB2V1_HUMAN]	4.91E-09	0.67550832
P61088	Ubiquitin-conjugating enzyme E2 N OS=Homo sapiens GN=UBE2N PE=1 SV=1 - [UBE2N_HUMAN]	0.00000439	0.792218652
P68036	Ubiquitin-conjugating enzyme E2 L3 OS=Homo sapiens GN=UBE2L3 PE=1 SV=1 - [UB2L3_HUMAN]	1.91E-08	0.777895792
P62979	Ubiquitin-40S ribosomal protein S27a OS=Homo sapiens GN=RPS27A PE=1 SV=2 - [RS27A_HUMAN]	1.23E-09	0.300247355
Q96FW1	Ubiquitin thioesterase OTUB1 OS=Homo sapiens GN=OTUB1	0.01364	0.72605

	PE=1 SV=2 - [OTUB1_HUMAN]	0037	823
P09936	Ubiquitin carboxyl-terminal hydrolase isozyme L1 OS=Homo sapiens GN=UCHL1 PE=1 SV=2 - [UCHL1_HUMAN]	7.76E-13	0.699017696
O43290	U4/U6.U5 tri-snRNP-associated protein 1 OS=Homo sapiens GN=SART1 PE=1 SV=1 - [SNUT1_HUMAN]	0.007150536	1.289075089
O43399	Tumor protein D54 OS=Homo sapiens GN=TPD52L2 PE=1 SV=2 - [TPD54_HUMAN]	0.002634539	0.762340385
P07951	Tropomyosin beta chain OS=Homo sapiens GN=TPM2 PE=1 SV=1 - [TPM2_HUMAN]	3.29E-09	0.443554157
P67936	Tropomyosin alpha-4 chain OS=Homo sapiens GN=TPM4 PE=1 SV=3 - [TPM4_HUMAN]	7.48E-32	0.642343268
P06753	Tropomyosin alpha-3 chain OS=Homo sapiens GN=TPM3 PE=1 SV=2 - [TPM3_HUMAN]	4.84E-10	0.735056633
P09493	Tropomyosin alpha-1 chain OS=Homo sapiens GN=TPM1 PE=1 SV=2 - [TPM1_HUMAN]	4.86E-27	0.316501715
P60174	Triosephosphate isomerase OS=Homo sapiens GN=TPI1 PE=1 SV=3 - [TPIS_HUMAN]	2.96E-97	0.691311607
P55084	Trifunctional enzyme subunit beta, mitochondrial OS=Homo sapiens GN=HADHB PE=1 SV=3 - [ECHB_HUMAN]	1.63E-08	1.205106287
P40939	Trifunctional enzyme subunit alpha, mitochondrial OS=Homo sapiens GN=HADHA PE=1 SV=2 - [ECHA_HUMAN]	3.33E-23	1.21881815
P53007	Tricarboxylate transport protein, mitochondrial OS=Homo sapiens GN=SLC25A1 PE=1 SV=2 - [TXTP_HUMAN]	0.000643927	1.274929423
P02766	Transthyretin OS=Homo sapiens GN=TTR PE=1 SV=1 - [TTHY_HUMAN]	0.0000556	1.336267883
Q6UW68	Transmembrane protein 205 OS=Homo sapiens GN=TMEM205 PE=1 SV=1 - [TM205_HUMAN]	3.18E-08	1.454369168
Q9BVC6	Transmembrane protein 109 OS=Homo sapiens GN=TMEM109 PE=1 SV=1 - [TM109_HUMAN]	0.00206945	1.42993327
Q9UM00	Transmembrane and coiled-coil domain-containing protein 1 OS=Homo sapiens GN=TMCO1 PE=1 SV=1 - [TMCO1_HUMAN]	0.000279301	1.335113485
Q9HD45	Transmembrane 9 superfamily member 3 OS=Homo sapiens GN=TM9SF3 PE=1 SV=2 - [TM9S3_HUMAN]	0.004858944	1.295057198
Q9UNL2	Translocon-associated protein subunit gamma OS=Homo sapiens GN=SSR3 PE=1 SV=1 - [SSRG_HUMAN]	0.010624693	1.258059443
P51571	Translocon-associated protein subunit delta OS=Homo sapiens GN=SSR4 PE=1 SV=1 - [SSRD_HUMAN]	0.001077725	1.333629696
P43307	Translocon-associated protein subunit alpha OS=Homo sapiens GN=SSR1 PE=1 SV=3 - [SSRA_HUMAN]	6.99E-11	1.32837407
P30536	Translocator protein OS=Homo sapiens GN=TSPO PE=1 SV=3 - [TSPOA_HUMAN]	0.000155767	1.259366539

Q9UGP8	Translocation protein SEC63 homolog OS=Homo sapiens GN=SEC63 PE=1 SV=2 - [SEC63_HUMAN]	0.00951 3993	1.22824 9744
P29401	Transketolase OS=Homo sapiens GN=TKT PE=1 SV=3 - [TKT_HUMAN]	2.99E-6 8	0.71493 749
P55072	Transitional endoplasmic reticulum ATPase OS=Homo sapiens GN=VCP PE=1 SV=4 - [TERA_HUMAN]	4.32E-3 5	0.74680 5927
Q9UI15	Transgelin-3 OS=Homo sapiens GN=TAGLN3 PE=1 SV=2 - [TAGL3_HUMAN]	0.00013 3681	0.70915 699
P37802	Transgelin-2 OS=Homo sapiens GN=TAGLN2 PE=1 SV=3 - [TAGL2_HUMAN]	5.41E-3 8	0.57589 8844
Q01995	Transgelin OS=Homo sapiens GN=TAGLN PE=1 SV=4 - [TAGL_HUMAN]	1.65E-1 19	0.43693 7614
P61812	Transforming growth factor beta-2 OS=Homo sapiens GN=TGFB2 PE=1 SV=1 - [TGFB2_HUMAN]	2.08E-0 8	0.78102 5198
P62995	Transformer-2 protein homolog beta OS=Homo sapiens GN=TRA2B PE=1 SV=1 - [TRA2B_HUMAN]	0.00309 9293	1.47956 3529
P02786	Transferrin receptor protein 1 OS=Homo sapiens GN=TFRC PE=1 SV=2 - [TFR1_HUMAN]	3.26E-0 9	1.22347 1836
Q15370	Transcription elongation factor B polypeptide 2 OS=Homo sapiens GN=TCEB2 PE=1 SV=1 - [ELOB_HUMAN]	0.00712 0873	0.74077 9936
P37837	Transaldolase OS=Homo sapiens GN=TALDO1 PE=1 SV=2 - [TALDO_HUMAN]	0.00000 488	0.76954 2325
P04066	Tissue alpha-L-fucosidase OS=Homo sapiens GN=FUCA1 PE=1 SV=4 - [FUCO_HUMAN]	0.00041 7467	0.65238 6648
P62328	Thymosin beta-4 OS=Homo sapiens GN=TMSB4X PE=1 SV=2 - [TYB4_HUMAN]	0.00000 0214	0.70272 0112
P19971	Thymidine phosphorylase OS=Homo sapiens GN=TYMP PE=1 SV=2 - [TYPH_HUMAN]	0.01965 7696	1.56006 2402
P07996	Thrombospondin-1 OS=Homo sapiens GN=THBS1 PE=1 SV=2 - [TSP1_HUMAN]	9.09E-1 30	0.48710 8107
Q9H3N1	Thioredoxin-related transmembrane protein 1 OS=Homo sapiens GN=TMX1 PE=1 SV=1 - [TMX1_HUMAN]	0.00001 72	1.31839 1562
P30048	Thioredoxin-dependent peroxide reductase, mitochondrial OS=Homo sapiens GN=PRDX3 PE=1 SV=3 - [PRDX3_HUMAN]	7.62E-1 0	0.75488 4547
P10599	Thioredoxin OS=Homo sapiens GN=TXN PE=1 SV=3 - [THIO_HUMAN]	0.01724 9815	0.79686 5662
Q8NBS9	Thioredoxin domain-containing protein 5 OS=Homo sapiens GN=TXNDC5 PE=1 SV=2 - [TXND5_HUMAN]	1.78E-2 0	0.61982 5045
O95881	Thioredoxin domain-containing protein 12 OS=Homo sapiens GN=TXNDC12 PE=1 SV=1 - [TXD12_HUMAN]	0.00059 3702	0.78346 2906
Q9Y490	Talin-1 OS=Homo sapiens GN=TLN1 PE=1 SV=3 -	1.24E-2	0.52733

	[TLN1_HUMAN]	0	2461
P04179	Superoxide dismutase [Mn], mitochondrial OS=Homo sapiens GN=SOD2 PE=1 SV=2 - [SODM_HUMAN]	4.44E-23	1.522379869
P00441	Superoxide dismutase [Cu-Zn] OS=Homo sapiens GN=SOD1 PE=1 SV=2 - [SODC_HUMAN]	0.0000203	0.790722193
P50225	Sulfotransferase 1A1 OS=Homo sapiens GN=SULT1A1 PE=1 SV=3 - [ST1A1_HUMAN]	0.000574122	1.387443635
Q9Y6N5	Sulfide:quinone oxidoreductase, mitochondrial OS=Homo sapiens GN=SQRDL PE=1 SV=1 - [SQRD_HUMAN]	1.98E-10	1.430546946
P38646	Stress-70 protein, mitochondrial OS=Homo sapiens GN=HSPA9 PE=1 SV=2 - [GRP75_HUMAN]	3.73E-18	1.218500905
Q9UJZ1	Stomatin-like protein 2, mitochondrial OS=Homo sapiens GN=STOML2 PE=1 SV=1 - [STML2_HUMAN]	1.88E-08	1.412073227
Q15738	Sterol-4-alpha-carboxylate 3-dehydrogenase, decarboxylating OS=Homo sapiens GN=NSDHL PE=1 SV=2 - [NSDHL_HUMAN]	0.000574028	1.298490505
Q14247	Src substrate cortactin OS=Homo sapiens GN=CTTN PE=1 SV=2 - [SRC8_HUMAN]	0.0000015	0.784769656
Q12874	Splicing factor 3A subunit 3 OS=Homo sapiens GN=SF3A3 PE=1 SV=1 - [SF3A3_HUMAN]	0.013603896	0.690965624
P09486	SPARC OS=Homo sapiens GN=SPARC PE=1 SV=1 - [SPRC_HUMAN]	1.14E-08	0.401860038
O94875	Sorbin and SH3 domain-containing protein 2 OS=Homo sapiens GN=SORBS2 PE=1 SV=3 - [SRBS2_HUMAN]	0.000178404	0.770935723
P11166	Solute carrier family 2, facilitated glucose transporter member 1 OS=Homo sapiens GN=SLC2A1 PE=1 SV=2 - [GTR1_HUMAN]	7.85E-17	1.305331859
P54709	Sodium/potassium-transporting ATPase subunit beta-3 OS=Homo sapiens GN=ATP1B3 PE=1 SV=1 - [AT1B3_HUMAN]	0.006944261	1.233349778
P61956	Small ubiquitin-related modifier 2 OS=Homo sapiens GN=SUMO2 PE=1 SV=3 - [SUMO2_HUMAN]	0.0000394	0.727581398
P42226	Signal transducer and activator of transcription 6 OS=Homo sapiens GN=STAT6 PE=1 SV=1 - [STAT6_HUMAN]	0.010032111	1.206272618
Q9H9B4	Sideroflexin-1 OS=Homo sapiens GN=SFXN1 PE=1 SV=4 - [SFXN1_HUMAN]	0.008011694	1.349831272
P50454	Serpin H1 OS=Homo sapiens GN=SERPINH1 PE=1 SV=2 - [SERPH_HUMAN]	1.41E-107	0.683253128
P02787	Serotransferrin OS=Homo sapiens GN=TF PE=1 SV=3 - [TRFE_HUMAN]	0.045747523	1.293382194
P34897	Serine hydroxymethyltransferase, mitochondrial OS=Homo sapiens GN=SHMT2 PE=1 SV=3 - [GLYM_HUMAN]	1.01E-08	1.221467288
P83111	Serine beta-lactamase-like protein LACTB, mitochondrial OS=Homo sapiens GN=LACTB PE=1 SV=2 -	0.00846748	1.370801919

	[LACTB_HUMAN]		
Q8N474	Secreted frizzled-related protein 1 OS=Homo sapiens GN=SFRP1 PE=1 SV=1 - [SFRP1_HUMAN]	0.00000636	0.64112458
O76054	SEC14-like protein 2 OS=Homo sapiens GN=SEC14L2 PE=1 SV=1 - [S14L2_HUMAN]	0.00000299	0.727449078
P35637	RNA-binding protein FUS OS=Homo sapiens GN=FUS PE=1 SV=1 - [FUS_HUMAN]	0.036766045	0.754147813
Q01844	RNA-binding protein EWS OS=Homo sapiens GN=EWSR1 PE=1 SV=1 - [EWS_HUMAN]	0.00455472	0.770119369
P38159	RNA-binding motif protein, X chromosome OS=Homo sapiens GN=RBMX PE=1 SV=3 - [RBMX_HUMAN]	2.35E-09	0.794596742
Q9P2E9	Ribosome-binding protein 1 OS=Homo sapiens GN=RRBP1 PE=1 SV=4 - [RRBP1_HUMAN]	3.06E-24	0.681272054
P60891	Ribose-phosphate pyrophosphokinase 1 OS=Homo sapiens GN=PRPS1 PE=1 SV=2 - [PRPS1_HUMAN]	0.01655312	0.743678731
Q07960	Rho GTPase-activating protein 1 OS=Homo sapiens GN=ARHGAP1 PE=1 SV=1 - [RHG01_HUMAN]	1.14E-08	0.79169331
P52565	Rho GDP-dissociation inhibitor 1 OS=Homo sapiens GN=ARHGDIA PE=1 SV=3 - [GDIR1_HUMAN]	1.45E-08	0.739228386
P09455	Retinol-binding protein 1 OS=Homo sapiens GN=RBP1 PE=1 SV=2 - [RET1_HUMAN]	1.11E-27	0.671107159
Q9NQC3	Reticulon-4 OS=Homo sapiens GN=RTN4 PE=1 SV=2 - [RTN4_HUMAN]	5.59E-10	0.528041334
Q96D15	Reticulocalbin-3 OS=Homo sapiens GN=RCN3 PE=1 SV=1 - [RCN3_HUMAN]	0.043676488	0.701139351
Q00765	Receptor expression-enhancing protein 5 OS=Homo sapiens GN=REEP5 PE=1 SV=3 - [REEP5_HUMAN]	0.000401296	0.776884692
Q15404	Ras suppressor protein 1 OS=Homo sapiens GN=RSU1 PE=1 SV=3 - [RSU1_HUMAN]	0.000000515	0.712280555
O43504	Ragulator complex protein LAMTOR5 OS=Homo sapiens GN=LAMTOR5 PE=1 SV=1 - [LTOR5_HUMAN]	0.00067735	0.738461538
P35241	Radixin OS=Homo sapiens GN=RDX PE=1 SV=1 - [RADI_HUMAN]	0.011889521	0.758917278
P50395	Rab GDP dissociation inhibitor beta OS=Homo sapiens GN=GDI2 PE=1 SV=2 - [GDIB_HUMAN]	0.000000554	0.786239431
P14618	Pyruvate kinase PKM OS=Homo sapiens GN=PKM PE=1 SV=4 - [KPYM_HUMAN]	6.1E-152	0.619481815
A8MWD9	Putative small nuclear ribonucleoprotein G-like protein 15 OS=Homo sapiens GN=SNRPGP15 PE=5 SV=2 - [RUXGL_HUMAN]	0.013660598	0.783852636
P55786	Puromycin-sensitive aminopeptidase OS=Homo sapiens GN=NPEPPS PE=1 SV=2 - [PSA_HUMAN]	1.33E-09	0.797366646

P00734	Prothrombin OS=Homo sapiens GN=F2 PE=1 SV=2 - [THRB_HUMAN]	2.86E-27	1.836448093
P22061	Protein-L-isoaspartate(D-aspartate) O-methyltransferase OS=Homo sapiens GN=PCMT1 PE=1 SV=4 - [PIMT_HUMAN]	0.000500635	0.779473855
P21980	Protein-glutamine gamma-glutamyltransferase 2 OS=Homo sapiens GN=TGM2 PE=1 SV=2 - [TGM2_HUMAN]	2.09E-53	0.499356538
O94855	Protein transport protein Sec24D OS=Homo sapiens GN=SEC24D PE=1 SV=2 - [SC24D_HUMAN]	0.0000447	0.792124024
Q01105	Protein SET OS=Homo sapiens GN=SET PE=1 SV=3 - [SET_HUMAN]	1.72E-08	0.734861846
P06703	Protein S100-A6 OS=Homo sapiens GN=S100A6 PE=1 SV=1 - [S10A6_HUMAN]	0.0000492	0.761490345
Q96FQ6	Protein S100-A16 OS=Homo sapiens GN=S100A16 PE=1 SV=1 - [S10AG_HUMAN]	0.001229136	0.783852636
Q9Y570	Protein phosphatase methylesterase 1 OS=Homo sapiens GN=PPME1 PE=1 SV=3 - [PPME1_HUMAN]	2.72E-09	0.668197954
Q15435	Protein phosphatase 1 regulatory subunit 7 OS=Homo sapiens GN=PPP1R7 PE=1 SV=1 - [PP1R7_HUMAN]	0.001555852	0.681379415
O60237	Protein phosphatase 1 regulatory subunit 12B OS=Homo sapiens GN=PPP1R12B PE=1 SV=2 - [MYPT2_HUMAN]	0.035564577	0.740603592
Q86UE4	Protein LYRIC OS=Homo sapiens GN=MTDH PE=1 SV=2 - [LYRIC_HUMAN]	0.001509874	1.230958609
P07237	Protein disulfide-isomerase OS=Homo sapiens GN=P4HB PE=1 SV=3 - [PDIA1_HUMAN]	3.29E-177	0.698006069
Q15084	Protein disulfide-isomerase A6 OS=Homo sapiens GN=PDIA6 PE=1 SV=1 - [PDIA6_HUMAN]	8.76E-43	0.638875579
P13667	Protein disulfide-isomerase A4 OS=Homo sapiens GN=PDIA4 PE=1 SV=2 - [PDIA4_HUMAN]	1.25E-37	0.759798092
P30101	Protein disulfide-isomerase A3 OS=Homo sapiens GN=PDIA3 PE=1 SV=4 - [PDIA3_HUMAN]	1.75E-99	0.602087236
Q99497	Protein deglycase DJ-1 OS=Homo sapiens GN=PARK7 PE=1 SV=2 - [PARK7_HUMAN]	3.59E-22	0.710707154
Q9BT09	Protein canopy homolog 3 OS=Homo sapiens GN=CNPY3 PE=1 SV=1 - [CNPY3_HUMAN]	0.01100543	0.79586152
P41223	Protein BUD31 homolog OS=Homo sapiens GN=BUD31 PE=1 SV=2 - [BUD31_HUMAN]	0.049819407	0.691921813
Q96IU4	Protein ABHD14B OS=Homo sapiens GN=ABHD14B PE=1 SV=1 - [ABHEB_HUMAN]	0.000258206	0.779980501
P49720	Proteasome subunit beta type-3 OS=Homo sapiens GN=PSMB3 PE=1 SV=2 - [PSB3_HUMAN]	0.049435648	0.796284008
P49721	Proteasome subunit beta type-2 OS=Homo sapiens GN=PSMB2 PE=1 SV=1 - [PSB2_HUMAN]	0.049402029	0.769526741

P07602	Prosaposin OS=Homo sapiens GN=PSAP PE=1 SV=2 - [SAP_HUMAN]	1.67E-64	0.559337526
Q14005	Pro-interleukin-16 OS=Homo sapiens GN=IL16 PE=1 SV=4 - [IL16_HUMAN]	0.000011	0.675789829
Q99623	Prohibitin-2 OS=Homo sapiens GN=PHB2 PE=1 SV=2 - [PHB2_HUMAN]	9.73E-13	1.261829653
P35232	Prohibitin OS=Homo sapiens GN=PHB PE=1 SV=1 - [PHB_HUMAN]	1.01E-32	1.37779646
P07737	Profilin-1 OS=Homo sapiens GN=PFN1 PE=1 SV=2 - [PROF1_HUMAN]	2.91E-32	0.733602546
Q96IZ0	PRKC apoptosis WT1 regulator protein OS=Homo sapiens GN=PAWR PE=1 SV=1 - [PAWR_HUMAN]	0.000000361	0.594177065
P61758	Prefoldin subunit 3 OS=Homo sapiens GN=VBP1 PE=1 SV=3 - [PFD3_HUMAN]	0.000977494	0.679908698
P0CG39	POTE ankyrin domain family member J OS=Homo sapiens GN=POTEJ PE=3 SV=1 - [POTEJ_HUMAN]	3.15E-08	0.736210216
P26599	Polypyrimidine tract-binding protein 1 OS=Homo sapiens GN=PTBP1 PE=1 SV=1 - [PTBP1_HUMAN]	0.009862934	0.794060428
Q9UHX1	Poly(U)-binding-splicing factor PUF60 OS=Homo sapiens GN=PUF60 PE=1 SV=1 - [PUF60_HUMAN]	0.045064546	0.799223611
Q9UKK3	Poly [ADP-ribose] polymerase 4 OS=Homo sapiens GN=PARP4 PE=1 SV=3 - [PARP4_HUMAN]	0.028105222	0.650512278
P13797	Plastin-3 OS=Homo sapiens GN=PLS3 PE=1 SV=4 - [PLST_HUMAN]	9.43E-16	0.796481629
Q9HBL7	Plasminogen receptor (KT) OS=Homo sapiens GN=PLGRKT PE=1 SV=1 - [PLRKT_HUMAN]	0.02389158	1.221374046
P00747	Plasminogen OS=Homo sapiens GN=PLG PE=1 SV=2 - [PLMN_HUMAN]	1.83E-13	2.080443827
Q8NC51	Plasminogen activator inhibitor 1 RNA-binding protein OS=Homo sapiens GN=SERBP1 PE=1 SV=2 - [PAIRB_HUMAN]	0.000036	0.770008181
P05155	Plasma protease C1 inhibitor OS=Homo sapiens GN=SERPING1 PE=1 SV=2 - [IC1_HUMAN]	0.019564904	1.746724891
P23634	Plasma membrane calcium-transporting ATPase 4 OS=Homo sapiens GN=ATP2B4 PE=1 SV=2 - [AT2B4_HUMAN]	0.000243347	1.351210893
Q9H008	Phospholysine phosphohistidine inorganic pyrophosphate phosphatase OS=Homo sapiens GN=LHPP PE=1 SV=2 - [LHPP_HUMAN]	0.000114827	0.781504396
Q8IV08	Phospholipase D3 OS=Homo sapiens GN=PLD3 PE=1 SV=1 - [PLD3_HUMAN]	0.000919397	0.716864231
P18669	Phosphoglycerate mutase 1 OS=Homo sapiens GN=PGAM1 PE=1 SV=2 - [PGAM1_HUMAN]	2.83E-48	0.708345416
P07205	Phosphoglycerate kinase 2 OS=Homo sapiens GN=PGK2 PE=1	0.02699	0.79428

	SV=3 - [PGK2_HUMAN]	1311	1176
P00558	Phosphoglycerate kinase 1 OS=Homo sapiens GN=PGK1 PE=1 SV=3 - [PGK1_HUMAN]	1.21E-58	0.646681027
Q15124	Phosphoglucomutase-like protein 5 OS=Homo sapiens GN=PGM5 PE=1 SV=2 - [PGM5_HUMAN]	1.17E-08	0.668049647
Q13492	Phosphatidylinositol-binding clathrin assembly protein OS=Homo sapiens GN=PICALM PE=1 SV=2 - [PICAL_HUMAN]	0.001031229	0.78057919
P30086	Phosphatidylethanolamine-binding protein 1 OS=Homo sapiens GN=PEBP1 PE=1 SV=3 - [PEBP1_HUMAN]	2.05E-16	0.766449747
O95674	Phosphatidate cytidylyltransferase 2 OS=Homo sapiens GN=CDS2 PE=1 SV=1 - [CDS2_HUMAN]	0.033535997	1.433349259
O95571	Persulfide dioxygenase ETHE1, mitochondrial OS=Homo sapiens GN=ETHE1 PE=1 SV=2 - [ETHE1_HUMAN]	0.010496984	1.238517906
P51659	Peroxisomal multifunctional enzyme type 2 OS=Homo sapiens GN=HSD17B4 PE=1 SV=3 - [DHB4_HUMAN]	0.00118144	1.283027946
Q15067	Peroxisomal acyl-coenzyme A oxidase 1 OS=Homo sapiens GN=ACOX1 PE=1 SV=3 - [ACOX1_HUMAN]	0.04103722	1.494396014
P30041	Peroxiredoxin-6 OS=Homo sapiens GN=PRDX6 PE=1 SV=3 - [PRDX6_HUMAN]	9.7E-15	0.516932408
P32119	Peroxiredoxin-2 OS=Homo sapiens GN=PRDX2 PE=1 SV=5 - [PRDX2_HUMAN]	2.35E-21	0.766462027
Q99541	Perilipin-2 OS=Homo sapiens GN=PLIN2 PE=1 SV=2 - [PLIN2_HUMAN]	0.000214342	2.444987775
Q13526	Peptidyl-prolyl cis-trans isomerase NIMA-interacting 1 OS=Homo sapiens GN=PIN1 PE=1 SV=1 - [PIN1_HUMAN]	0.032471858	0.791702953
P26885	Peptidyl-prolyl cis-trans isomerase FKBP2 OS=Homo sapiens GN=FKBP2 PE=1 SV=2 - [FKBP2_HUMAN]	7.72E-14	0.719395707
P45877	Peptidyl-prolyl cis-trans isomerase C OS=Homo sapiens GN=PPIC PE=1 SV=1 - [PPIC_HUMAN]	0.00000427	0.589829652
P23284	Peptidyl-prolyl cis-trans isomerase B OS=Homo sapiens GN=PPIB PE=1 SV=2 - [PPIB_HUMAN]	2.69E-85	0.457231239
P62937	Peptidyl-prolyl cis-trans isomerase A OS=Homo sapiens GN=PPIA PE=1 SV=2 - [PPIA_HUMAN]	5.02E-49	0.70608928
P26022	Pentraxin-related protein PTX3 OS=Homo sapiens GN=PTX3 PE=1 SV=3 - [PTX3_HUMAN]	4.42E-08	2.324005034
Q9NR12	PDZ and LIM domain protein 7 OS=Homo sapiens GN=PDLIM7 PE=1 SV=1 - [PDLI7_HUMAN]	5.59E-14	0.777926049
Q96HC4	PDZ and LIM domain protein 5 OS=Homo sapiens GN=PDLIM5 PE=1 SV=5 - [PDLI5_HUMAN]	2.01E-16	0.76271071
P50479	PDZ and LIM domain protein 4 OS=Homo sapiens GN=PDLIM4 PE=1 SV=2 - [PDLI4_HUMAN]	6.22E-10	0.798420901
Q8WX93	Palladin OS=Homo sapiens GN=PALLD PE=1 SV=3 -	8.5E-12	0.75884

	[PALLD_HUMAN]		6064
Q9Y3B8	Oligoribonuclease, mitochondrial OS=Homo sapiens GN=REXO2 PE=1 SV=3 - [ORN_HUMAN]	0.01231 9487	0.66146 3157
P55209	Nucleosome assembly protein 1-like 1 OS=Homo sapiens GN=NAP1L1 PE=1 SV=1 - [NP1L1_HUMAN]	0.00015 4578	0.79010 5449
P22392	Nucleoside diphosphate kinase B OS=Homo sapiens GN=NME2 PE=1 SV=1 - [NDKB_HUMAN]	1.09E-1 8	0.64548 2062
P19338	Nucleolin OS=Homo sapiens GN=NCL PE=1 SV=3 - [NUCL_HUMAN]	4.5E-16	0.78496 3587
P80303	Nucleobindin-2 OS=Homo sapiens GN=NUCB2 PE=1 SV=2 - [NUCB2_HUMAN]	0.00003 57	0.73893 7668
P43490	Nicotinamide phosphoribosyltransferase OS=Homo sapiens GN=NAMPT PE=1 SV=1 - [NAMPT_HUMAN]	1.07E-0 8	1.39778 6837
Q0ZGT2	Nexilin OS=Homo sapiens GN=NEXN PE=1 SV=1 - [NEXN_HUMAN]	0.00013 9489	0.70571 6302
Q09666	Neuroblast differentiation-associated protein AHNAK OS=Homo sapiens GN=AHNAK PE=1 SV=2 - [AHNK_HUMAN]	3.45E-1 36	0.74205 1881
Q8TBC4	NEDD8-activating enzyme E1 catalytic subunit OS=Homo sapiens GN=UBA3 PE=1 SV=2 - [UBA3_HUMAN]	0.00019 8503	1.30633 5728
Q15843	NEDD8 OS=Homo sapiens GN=NEDD8 PE=1 SV=1 - [NEDD8_HUMAN]	0.00000 0267	0.72194 3472
Q13765	Nascent polypeptide-associated complex subunit alpha OS=Homo sapiens GN=NACA PE=1 SV=1 - [NACA_HUMAN]	3.08E-0 8	0.76120 27
O75489	NADH dehydrogenase [ubiquinone] iron-sulfur protein 3, mitochondrial OS=Homo sapiens GN=NDUFS3 PE=1 SV=1 - [NDUS3_HUMAN]	0.00643 5198	1.26490 7842
P49821	NADH dehydrogenase [ubiquinone] flavoprotein 1, mitochondrial OS=Homo sapiens GN=NDUFV1 PE=1 SV=4 - [NDUV1_HUMAN]	0.00003 75	1.35104 7061
O96000	NADH dehydrogenase [ubiquinone] 1 beta subcomplex subunit 10 OS=Homo sapiens GN=NDUFB10 PE=1 SV=3 - [NDUBA_HUMAN]	0.00915 4978	1.32625 9947
O75438	NADH dehydrogenase [ubiquinone] 1 beta subcomplex subunit 1 OS=Homo sapiens GN=NDUFB1 PE=1 SV=1 - [NDUB1_HUMAN]	0.00000 445	1.34907 2513
Q16795	NADH dehydrogenase [ubiquinone] 1 alpha subcomplex subunit 9, mitochondrial OS=Homo sapiens GN=NDUFA9 PE=1 SV=2 - [NDUA9_HUMAN]	0.00000 142	1.31771 5959
Q9P0J0	NADH dehydrogenase [ubiquinone] 1 alpha subcomplex subunit 13 OS=Homo sapiens GN=NDUFA13 PE=1 SV=3 - [NDUAD_HUMAN]	0.00007 17	1.29198 9664
O95299	NADH dehydrogenase [ubiquinone] 1 alpha subcomplex subunit	0.00161	1.22100

	10, mitochondrial OS=Homo sapiens GN=NDUFA10 PE=1 SV=1 - [NDUAA_HUMAN]	4226	1221
Q13423	NAD(P) transhydrogenase, mitochondrial OS=Homo sapiens GN=NNT PE=1 SV=3 - [NNTM_HUMAN]	1.15E-14	1.263479564
O14745	Na(+)/H(+) exchange regulatory cofactor NHE-RF1 OS=Homo sapiens GN=SLC9A3R1 PE=1 SV=4 - [NHRF1_HUMAN]	0.000000137	0.77789062
P29966	Myristoylated alanine-rich C-kinase substrate OS=Homo sapiens GN=MARCKS PE=1 SV=4 - [MARCS_HUMAN]	5.71E-14	0.510066309
P58546	Myotrophin OS=Homo sapiens GN=MTPN PE=1 SV=2 - [MTPN_HUMAN]	0.000000329	0.738916256
P35579	Myosin-9 OS=Homo sapiens GN=MYH9 PE=1 SV=4 - [MYH9_HUMAN]	1.09E-81	0.654047502
P60660	Myosin light polypeptide 6 OS=Homo sapiens GN=MYL6 PE=1 SV=2 - [MYL6_HUMAN]	2.32E-13	0.779880567
Q9NZM1	Myoferlin OS=Homo sapiens GN=MYOF PE=1 SV=1 - [MYOF_HUMAN]	2.84E-16	1.231775562
Q969H8	Myeloid-derived growth factor OS=Homo sapiens GN=MYDGF PE=1 SV=1 - [MYDGF_HUMAN]	7.86E-17	0.583196647
Q96S97	Myeloid-associated differentiation marker OS=Homo sapiens GN=MYADM PE=1 SV=2 - [MYADM_HUMAN]	0.01064793	1.284384031
Q6UB35	Monofunctional C1-tetrahydrofolate synthase, mitochondrial OS=Homo sapiens GN=MTHFD1L PE=1 SV=1 - [C1TM_HUMAN]	0.013627382	1.362212233
P26038	Moesin OS=Homo sapiens GN=MSN PE=1 SV=3 - [MOES_HUMAN]	3.05E-28	0.733900737
P27361	Mitogen-activated protein kinase 3 OS=Homo sapiens GN=MAPK3 PE=1 SV=4 - [MK03_HUMAN]	2.28E-09	1.294498382
Q9Y3D6	Mitochondrial fission 1 protein OS=Homo sapiens GN=FIS1 PE=1 SV=2 - [FIS1_HUMAN]	0.000143431	0.726282342
Q9Y6C9	Mitochondrial carrier homolog 2 OS=Homo sapiens GN=MTCH2 PE=1 SV=1 - [MTCH2_HUMAN]	3.31E-09	1.25782724
Q15691	Microtubule-associated protein RP/EB family member 1 OS=Homo sapiens GN=MAPRE1 PE=1 SV=3 - [MARE1_HUMAN]	4.95E-14	0.715227644
P27816	Microtubule-associated protein 4 OS=Homo sapiens GN=MAP4 PE=1 SV=3 - [MAP4_HUMAN]	1.7E-14	0.747225924
P10620	Microsomal glutathione S-transferase 1 OS=Homo sapiens GN=MGST1 PE=1 SV=1 - [MGST1_HUMAN]	0.0000587	1.328805551
P55001	Microfibrillar-associated protein 2 OS=Homo sapiens GN=MFAP2 PE=2 SV=1 - [MFAP2_HUMAN]	0.016033196	1.211061024
Q16891	MICOS complex subunit MIC60 OS=Homo sapiens GN=IMMT PE=1 SV=1 - [MIC60_HUMAN]	0.00000265	1.217263002

Q02252	Methylmalonate-semialdehyde dehydrogenase [acylating], mitochondrial OS=Homo sapiens GN=ALDH6A1 PE=1 SV=2 - [MMSA_HUMAN]	0.002608789	1.224489795
P84157	Matrix-remodeling-associated protein 7 OS=Homo sapiens GN=MXRA7 PE=1 SV=1 - [MXRA7_HUMAN]	8.47E-11	0.57176891
P40925	Malate dehydrogenase, cytoplasmic OS=Homo sapiens GN=MDH1 PE=1 SV=4 - [MDHC_HUMAN]	0.0000966	0.677171507
P14174	Macrophage migration inhibitory factor OS=Homo sapiens GN=MIF PE=1 SV=4 - [MIF_HUMAN]	0.0000022	0.732255871
P42785	Lysosomal Pro-X carboxypeptidase OS=Homo sapiens GN=PRCP PE=1 SV=1 - [PCP_HUMAN]	0.008157445	0.687468968
P10619	Lysosomal protective protein OS=Homo sapiens GN=CTSA PE=1 SV=2 - [PPGB_HUMAN]	0.002172894	0.772658912
P36776	Lon protease homolog, mitochondrial OS=Homo sapiens GN=LONP1 PE=1 SV=2 - [LONM_HUMAN]	4.2E-09	1.204128441
P07195	L-lactate dehydrogenase B chain OS=Homo sapiens GN=LDHB PE=1 SV=2 - [LDHB_HUMAN]	6.21E-25	0.686931135
P00338	L-lactate dehydrogenase A chain OS=Homo sapiens GN=LDHA PE=1 SV=2 - [LDHA_HUMAN]	2.89E-105	0.638798955
Q9UHB6	LIM domain and actin-binding protein 1 OS=Homo sapiens GN=LIMA1 PE=1 SV=1 - [LIMA1_HUMAN]	0.00000836	0.745446295
Q14847	LIM and SH3 domain protein 1 OS=Homo sapiens GN=LASP1 PE=1 SV=2 - [LASP1_HUMAN]	0.000042	0.508151598
Q9NZU5	LIM and cysteine-rich domains protein 1 OS=Homo sapiens GN=LMCD1 PE=1 SV=1 - [LMCD1_HUMAN]	0.0000443	0.695520218
P42704	Leucine-rich PPR motif-containing protein, mitochondrial OS=Homo sapiens GN=LRPPRC PE=1 SV=3 - [LPPRC_HUMAN]	4.1E-09	1.201923077
Q14767	Latent-transforming growth factor beta-binding protein 2 OS=Homo sapiens GN=LTBP2 PE=1 SV=3 - [LTBP2_HUMAN]	8.17E-21	0.684018399
P05787	Keratin, type II cytoskeletal 8 OS=Homo sapiens GN=KRT8 PE=1 SV=7 - [K2C8_HUMAN]	1.92E-60	0.351241241
P08729	Keratin, type II cytoskeletal 7 OS=Homo sapiens GN=KRT7 PE=1 SV=5 - [K2C7_HUMAN]	3.02E-98	0.597261338
P05783	Keratin, type I cytoskeletal 18 OS=Homo sapiens GN=KRT18 PE=1 SV=2 - [K1C18_HUMAN]	1.69E-87	0.434490166
P48735	Isocitrate dehydrogenase [NADP], mitochondrial OS=Homo sapiens GN=IDH2 PE=1 SV=2 - [IDHP_HUMAN]	1.93E-14	1.252348153
Q12906	Interleukin enhancer-binding factor 3 OS=Homo sapiens GN=ILF3 PE=1 SV=3 - [ILF3_HUMAN]	0.000171406	0.789144874
Q12905	Interleukin enhancer-binding factor 2 OS=Homo sapiens GN=ILF2 PE=1 SV=2 - [ILF2_HUMAN]	0.000000668	0.781157131

P09914	Interferon-induced protein with tetratricopeptide repeats 1 OS=Homo sapiens GN=IFIT1 PE=1 SV=2 - [IFIT1_HUMAN]	0.001453819	1.425661915
P20591	Interferon-induced GTP-binding protein Mx1 OS=Homo sapiens GN=MX1 PE=1 SV=4 - [MX1_HUMAN]	3.66E-09	1.530872598
P05362	Intercellular adhesion molecule 1 OS=Homo sapiens GN=ICAM1 PE=1 SV=2 - [ICAM1_HUMAN]	5.82E-18	1.726476753
Q06033	Inter-alpha-trypsin inhibitor heavy chain H3 OS=Homo sapiens GN=ITIH3 PE=1 SV=2 - [ITIH3_HUMAN]	0.000352699	1.718705243
P19823	Inter-alpha-trypsin inhibitor heavy chain H2 OS=Homo sapiens GN=ITIH2 PE=1 SV=2 - [ITIH2_HUMAN]	1.13E-20	1.822389354
P19827	Inter-alpha-trypsin inhibitor heavy chain H1 OS=Homo sapiens GN=ITIH1 PE=1 SV=3 - [ITIH1_HUMAN]	0.0000585	1.477013969
P08648	Integrin alpha-5 OS=Homo sapiens GN=ITGA5 PE=1 SV=2 - [ITA5_HUMAN]	0.00000645	1.238730989
P17301	Integrin alpha-2 OS=Homo sapiens GN=ITGA2 PE=1 SV=1 - [ITA2_HUMAN]	0.000000008	1.449835686
Q9UKX5	Integrin alpha-11 OS=Homo sapiens GN=ITGA11 PE=1 SV=2 - [ITA11_HUMAN]	0.003930281	0.678794461
Q16270	Insulin-like growth factor-binding protein 7 OS=Homo sapiens GN=IGFBP7 PE=1 SV=1 - [IBP7_HUMAN]	2.42E-12	0.503362579
P01308	Insulin OS=Homo sapiens GN=INS PE=1 SV=1 - [INS_HUMAN]	0.000144055	2.304811294
Q13308	Inactive tyrosine-protein kinase 7 OS=Homo sapiens GN=PTK7 PE=1 SV=2 - [PTK7_HUMAN]	0.0000113	0.747923178
P01871	Ig mu chain C region OS=Homo sapiens GN=IGHM PE=1 SV=3 - [IGHM_HUMAN]	0.005263739	1.368340032
P0CG05	Ig lambda-2 chain C regions OS=Homo sapiens GN=IGLC2 PE=1 SV=1 - [LAC2_HUMAN]	0.0000184	1.370520112
P01834	Ig kappa chain C region OS=Homo sapiens GN=IGKC PE=1 SV=1 - [IGKC_HUMAN]	1.13E-09	1.517322767
P01765	Ig heavy chain V-III region TIL OS=Homo sapiens PE=1 SV=1 - [HV304_HUMAN]	0.003725411	1.450326323
P01771	Ig heavy chain V-III region HIL OS=Homo sapiens PE=1 SV=1 - [HV310_HUMAN]	0.006395187	2.467612585
P01781	Ig heavy chain V-III region GAL OS=Homo sapiens PE=1 SV=1 - [HV320_HUMAN]	1.81E-08	3.070761014
P01768	Ig heavy chain V-III region CAM OS=Homo sapiens PE=1 SV=1 - [HV307_HUMAN]	0.006899594	1.87090739
P01767	Ig heavy chain V-III region BUT OS=Homo sapiens PE=1 SV=1 - [HV306_HUMAN]	0.002966083	1.997602877
P01766	Ig heavy chain V-III region BRO OS=Homo sapiens PE=1 SV=1 - [HV305_HUMAN]	5.77E-15	2.161263506

P06331	Ig heavy chain V-II region ARH-77 OS=Homo sapiens PE=4 SV=1 - [HV209_HUMAN]	2.59E-08	1.962901168
P01743	Ig heavy chain V-I region HG3 OS=Homo sapiens PE=3 SV=1 - [HV102_HUMAN]	7.83E-08	4.165135372
P01742	Ig heavy chain V-I region EU OS=Homo sapiens PE=1 SV=1 - [HV101_HUMAN]	4.26E-15	3.077799947
P01860	Ig gamma-3 chain C region OS=Homo sapiens GN=IGHG3 PE=1 SV=2 - [IGHG3_HUMAN]	0.00308589	1.684210526
P01859	Ig gamma-2 chain C region OS=Homo sapiens GN=IGHG2 PE=1 SV=2 - [IGHG2_HUMAN]	0.000036	1.639792294
P01857	Ig gamma-1 chain C region OS=Homo sapiens GN=IGHG1 PE=1 SV=1 - [IGHG1_HUMAN]	1.31E-18	1.689316013
P01876	Ig alpha-1 chain C region OS=Homo sapiens GN=IGHA1 PE=1 SV=2 - [IGHA1_HUMAN]	0.000240673	1.440144014
P35914	Hydroxymethylglutaryl-CoA lyase, mitochondrial OS=Homo sapiens GN=HMGCL PE=1 SV=2 - [HMGCL_HUMAN]	0.007396318	1.219512195
P10319	HLA class I histocompatibility antigen, B-58 alpha chain OS=Homo sapiens GN=HLA-B PE=1 SV=1 - [1B58_HUMAN]	0.001855983	1.429592566
Q71DI3	Histone H3.2 OS=Homo sapiens GN=HIST2H3A PE=1 SV=3 - [H32_HUMAN]	0.032286766	0.74190856
Q96KK5	Histone H2A type 1-H OS=Homo sapiens GN=HIST1H2AH PE=1 SV=3 - [H2A1H_HUMAN]	5.61E-13	0.736265402
P07305	Histone H1.0 OS=Homo sapiens GN=H1F0 PE=1 SV=3 - [H10_HUMAN]	0.014453814	1.46092038
P37235	Hippocalcin-like protein 1 OS=Homo sapiens GN=HPCAL1 PE=1 SV=3 - [HPCL1_HUMAN]	0.0000159	0.678569914
P09429	High mobility group protein B1 OS=Homo sapiens GN=HMGB1 PE=1 SV=3 - [HMGB1_HUMAN]	0.000493185	0.786898146
P22626	Heterogeneous nuclear ribonucleoproteins A2/B1 OS=Homo sapiens GN=HNRNPA2B1 PE=1 SV=2 - [ROA2_HUMAN]	2.64E-21	0.793993032
P09651	Heterogeneous nuclear ribonucleoprotein A1 OS=Homo sapiens GN=HNRNPA1 PE=1 SV=5 - [ROA1_HUMAN]	3.75E-19	0.711290496
Q99729	Heterogeneous nuclear ribonucleoprotein A/B OS=Homo sapiens GN=HNRNPAB PE=1 SV=2 - [ROAA_HUMAN]	0.001581272	0.609644577
P68871	Hemoglobin subunit beta OS=Homo sapiens GN=HBB PE=1 SV=2 - [HBB_HUMAN]	4.11E-15	1.501091703
P09601	Heme oxygenase 1 OS=Homo sapiens GN=HMOX1 PE=1 SV=1 - [HMOX1_HUMAN]	0.002377607	1.324064879
P08238	Heat shock protein HSP 90-beta OS=Homo sapiens GN=HSP90AB1 PE=1 SV=4 - [HS90B_HUMAN]	1.24E-14	0.710308119
P07900	Heat shock protein HSP 90-alpha OS=Homo sapiens GN=HSP90AA1 PE=1 SV=5 - [HS90A_HUMAN]	0.00000464	0.634112267

P04792	Heat shock protein beta-1 OS=Homo sapiens GN=HSPB1 PE=1 SV=2 - [HSPB1_HUMAN]	0.00000167	0.508843076
P11142	Heat shock cognate 71 kDa protein OS=Homo sapiens GN=HSPA8 PE=1 SV=1 - [HSP7C_HUMAN]	4.35E-32	0.777974481
Q53T59	HCLS1-binding protein 3 OS=Homo sapiens GN=HS1BP3 PE=1 SV=1 - [H1BP3_HUMAN]	0.00653166	1.206272618
P32455	Guanylate-binding protein 1 OS=Homo sapiens GN=GBP1 PE=1 SV=2 - [GBP1_HUMAN]	0.00000546	0.794706035
Q92963	GTP-binding protein Rit1 OS=Homo sapiens GN=RIT1 PE=1 SV=1 - [RIT1_HUMAN]	0.004384689	1.410437236
Q99988	Growth/differentiation factor 15 OS=Homo sapiens GN=GDF15 PE=2 SV=3 - [GDF15_HUMAN]	0.000241258	1.790510295
P28799	Granulins OS=Homo sapiens GN=GRN PE=1 SV=2 - [GRN_HUMAN]	0.000000189	0.720642813
Q9Y625	Glypican-6 OS=Homo sapiens GN=GPC6 PE=1 SV=1 - [GPC6_HUMAN]	0.027521936	0.784929356
P30419	Glycylpeptide N-tetradecanoyltransferase 1 OS=Homo sapiens GN=NMT1 PE=1 SV=2 - [NMT1_HUMAN]	0.022792422	0.786596397
P13807	Glycogen [starch] synthase, muscle OS=Homo sapiens GN=GYS1 PE=1 SV=2 - [GYS1_HUMAN]	0.00000733	1.279181324
P43304	Glycerol-3-phosphate dehydrogenase, mitochondrial OS=Homo sapiens GN=GPD2 PE=1 SV=3 - [GPDM_HUMAN]	0.001760959	1.612903226
P04406	Glyceraldehyde-3-phosphate dehydrogenase OS=Homo sapiens GN=GAPDH PE=1 SV=3 - [G3P_HUMAN]	8.39E-198	0.738325076
P09211	Glutathione S-transferase P OS=Homo sapiens GN=GSTP1 PE=1 SV=2 - [GSTP1_HUMAN]	7.77E-48	0.695353179
P22352	Glutathione peroxidase 3 OS=Homo sapiens GN=GPX3 PE=1 SV=2 - [GPX3_HUMAN]	0.000000772	1.32146605
Q86SX6	Glutaredoxin-related protein 5, mitochondrial OS=Homo sapiens GN=GLRX5 PE=1 SV=2 - [GLRX5_HUMAN]	0.001187385	1.351351351
P00367	Glutamate dehydrogenase 1, mitochondrial OS=Homo sapiens GN=GLUD1 PE=1 SV=2 - [DHE3_HUMAN]	0.000149528	1.262626263
P14314	Glucosidase 2 subunit beta OS=Homo sapiens GN=PRKCSH PE=1 SV=2 - [GLU2B_HUMAN]	1.49E-17	0.778785873
P06744	Glucose-6-phosphate isomerase OS=Homo sapiens GN=GPI PE=1 SV=4 - [G6PI_HUMAN]	1.93E-09	0.56453271
P07093	Glia-derived nexin OS=Homo sapiens GN=SERPINE2 PE=1 SV=1 - [GDN_HUMAN]	3.8E-12	1.250244188
Q92820	Gamma-glutamyl hydrolase OS=Homo sapiens GN=GGH PE=1 SV=2 - [GGH_HUMAN]	0.0000134	0.716127184
P09382	Galectin-1 OS=Homo sapiens GN=LGALS1 PE=1 SV=2 - [LEG1_HUMAN]	6.57E-36	0.75980446

P09972	Fructose-bisphosphate aldolase C OS=Homo sapiens GN=ALDOC PE=1 SV=2 - [ALDOC_HUMAN]	0.00000339	0.765567145
P04075	Fructose-bisphosphate aldolase A OS=Homo sapiens GN=ALDOA PE=1 SV=2 - [ALDOA_HUMAN]	1.21E-26	0.545075965
Q14192	Four and a half LIM domains protein 2 OS=Homo sapiens GN=FHL2 PE=1 SV=3 - [FHL2_HUMAN]	2.17E-09	0.793650794
Q12841	Follistatin-related protein 1 OS=Homo sapiens GN=FSTL1 PE=1 SV=1 - [FSTL1_HUMAN]	0.007376994	0.489436332
Q14315	Filamin-C OS=Homo sapiens GN=FLNC PE=1 SV=3 - [FLNC_HUMAN]	7.56E-35	0.751294896
O75369	Filamin-B OS=Homo sapiens GN=FLNB PE=1 SV=2 - [FLNB_HUMAN]	1.44E-106	0.736817973
P21333	Filamin-A OS=Homo sapiens GN=FLNA PE=1 SV=4 - [FLNA_HUMAN]	0	0.542326377
P98095	Fibulin-2 OS=Homo sapiens GN=FBLN2 PE=1 SV=2 - [FBLN2_HUMAN]	0.049890515	1.448051165
P23142	Fibulin-1 OS=Homo sapiens GN=FBLN1 PE=1 SV=4 - [FBLN1_HUMAN]	6.09E-10	0.741964524
P02751	Fibronectin OS=Homo sapiens GN=FN1 PE=1 SV=4 - [FINC_HUMAN]	0.000000355	0.692141886
P02679	Fibrinogen gamma chain OS=Homo sapiens GN=FGG PE=1 SV=3 - [FIBG_HUMAN]	2.99E-09	1.761245553
P02675	Fibrinogen beta chain OS=Homo sapiens GN=FGB PE=1 SV=2 - [FIBB_HUMAN]	1.34E-10	1.623849773
P02671	Fibrinogen alpha chain OS=Homo sapiens GN=FGA PE=1 SV=2 - [FIBA_HUMAN]	1.56E-11	1.655900581
P35556	Fibrillin-2 OS=Homo sapiens GN=FBN2 PE=1 SV=3 - [FBN2_HUMAN]	4.7E-54	1.364184899
P35555	Fibrillin-1 OS=Homo sapiens GN=FBN1 PE=1 SV=3 - [FBN1_HUMAN]	2.34E-56	1.232826873
P02794	Ferritin heavy chain OS=Homo sapiens GN=FTH1 PE=1 SV=2 - [FRIH_HUMAN]	0.000797876	1.205291228
Q5SYB0	FERM and PDZ domain-containing protein 1 OS=Homo sapiens GN=FRMPD1 PE=1 SV=1 - [FRPD1_HUMAN]	0.00000843	0.618085172
Q01469	Fatty acid-binding protein, epidermal OS=Homo sapiens GN=FABP5 PE=1 SV=3 - [FABP5_HUMAN]	0.006285083	0.770416025
P15311	Ezrin OS=Homo sapiens GN=EZR PE=1 SV=4 - [EZRI_HUMAN]	1.36E-20	0.508826297
Q14152	Eukaryotic translation initiation factor 3 subunit A OS=Homo sapiens GN=EIF3A PE=1 SV=1 - [EIF3A_HUMAN]	0.000178797	0.768049155
Q9BY44	Eukaryotic translation initiation factor 2A OS=Homo sapiens GN=EIF2A PE=1 SV=3 - [EIF2A_HUMAN]	0.011495148	0.764379897

Q14240	Eukaryotic initiation factor 4A-II OS=Homo sapiens GN=EIF4A2 PE=1 SV=2 - [IF4A2_HUMAN]	0.0000228	0.783972125
Q8N766	ER membrane protein complex subunit 1 OS=Homo sapiens GN=EMC1 PE=1 SV=1 - [EMC1_HUMAN]	0.0000444	1.30480167
P61916	Epididymal secretory protein E1 OS=Homo sapiens GN=NPC2 PE=1 SV=1 - [NPC2_HUMAN]	0.000766874	0.737463127
P17813	Endoglin OS=Homo sapiens GN=ENG PE=1 SV=2 - [EGLN_HUMAN]	0.036932727	1.576044129
Q9Y6C2	EMILIN-1 OS=Homo sapiens GN=EMILIN1 PE=1 SV=2 - [EMIL1_HUMAN]	0.003095527	1.391650099
P26641	Elongation factor 1-gamma OS=Homo sapiens GN=EEF1G PE=1 SV=3 - [EF1G_HUMAN]	1.59E-21	0.764747602
P29692	Elongation factor 1-delta OS=Homo sapiens GN=EEF1D PE=1 SV=5 - [EF1D_HUMAN]	3.98E-09	0.669812757
P24534	Elongation factor 1-beta OS=Homo sapiens GN=EEF1B2 PE=1 SV=3 - [EF1B_HUMAN]	3.68E-19	0.73981217
Q12805	EGF-containing fibulin-like extracellular matrix protein 1 OS=Homo sapiens GN=EFEMP1 PE=1 SV=2 - [FBLN3_HUMAN]	0.008896915	0.686829642
Q13561	Dynactin subunit 2 OS=Homo sapiens GN=DCTN2 PE=1 SV=4 - [DCTN2_HUMAN]	0.000000114	0.75234638
Q16643	Drebrin OS=Homo sapiens GN=DBN1 PE=1 SV=4 - [DREB_HUMAN]	5.91E-08	0.745606964
Q9Y673	Dolichyl-phosphate beta-glucosyltransferase OS=Homo sapiens GN=ALG5 PE=1 SV=1 - [ALG5_HUMAN]	0.00200351	1.315789474
Q8TCJ2	Dolichyl-diphosphooligosaccharide--protein glycosyltransferase subunit STT3B OS=Homo sapiens GN=STT3B PE=1 SV=1 - [STT3B_HUMAN]	0.002781709	1.210531626
Q16531	DNA damage-binding protein 1 OS=Homo sapiens GN=DDB1 PE=1 SV=1 - [DDB1_HUMAN]	0.005360782	0.782327631
Q12959	Disks large homolog 1 OS=Homo sapiens GN=DLG1 PE=1 SV=2 - [DLG1_HUMAN]	0.002419435	0.750308162
Q9UHL4	Dipeptidyl peptidase 2 OS=Homo sapiens GN=DPP7 PE=1 SV=3 - [DPP2_HUMAN]	0.000155641	0.52298406
O14531	Dihydropyrimidinase-related protein 4 OS=Homo sapiens GN=DPYSL4 PE=1 SV=2 - [DPYL4_HUMAN]	0.047098592	1.302295295
P36957	Dihydrolipoyllysine-residue succinyltransferase component of 2-oxoglutarate dehydrogenase complex, mitochondrial OS=Homo sapiens GN=DLST PE=1 SV=4 - [ODO2_HUMAN]	0.001359075	1.40039678
P54886	Delta-1-pyrroline-5-carboxylate synthase OS=Homo sapiens GN=ALDH18A1 PE=1 SV=2 - [P5CS_HUMAN]	0.00000109	1.206310911
O43175	D-3-phosphoglycerate dehydrogenase OS=Homo sapiens	0.00000	1.40944

	GN=PHGDH PE=1 SV=4 - [SERA_HUMAN]	11	327
P15954	Cytochrome c oxidase subunit 7C, mitochondrial OS=Homo sapiens GN=COX7C PE=1 SV=1 - [COX7C_HUMAN]	0.00044 6121	1.57697 6148
P14406	Cytochrome c oxidase subunit 7A2, mitochondrial OS=Homo sapiens GN=COX7A2 PE=1 SV=1 - [CX7A2_HUMAN]	0.00654 1051	1.26923 6871
P09669	Cytochrome c oxidase subunit 6C OS=Homo sapiens GN=COX6C PE=1 SV=2 - [COX6C_HUMAN]	0.00388 9319	1.41183 1145
P10606	Cytochrome c oxidase subunit 5B, mitochondrial OS=Homo sapiens GN=COX5B PE=1 SV=2 - [COX5B_HUMAN]	0.04749 392	1.20419 7489
P20674	Cytochrome c oxidase subunit 5A, mitochondrial OS=Homo sapiens GN=COX5A PE=1 SV=2 - [COX5A_HUMAN]	4.34E-0 8	1.52322 9246
P13073	Cytochrome c oxidase subunit 4 isoform 1, mitochondrial OS=Homo sapiens GN=COX4I1 PE=1 SV=1 - [COX41_HUMAN]	0.00000 0326	1.22966 8548
P00403	Cytochrome c oxidase subunit 2 OS=Homo sapiens GN=MT-CO2 PE=1 SV=1 - [COX2_HUMAN]	1.56E-1 4	1.36588 6973
Q9Y2R0	Cytochrome c oxidase assembly factor 3 homolog, mitochondrial OS=Homo sapiens GN=COA3 PE=1 SV=1 - [COA3_HUMAN]	0.00177 9469	1.27822 7525
P99999	Cytochrome c OS=Homo sapiens GN=CYCS PE=1 SV=2 - [CYC_HUMAN]	2.31E-1 7	0.52269 7574
P47985	Cytochrome b-c1 complex subunit Rieske, mitochondrial OS=Homo sapiens GN=UQCRFS1 PE=1 SV=2 - [UCRI_HUMAN]	0.00027 9557	1.26887 4508
P22695	Cytochrome b-c1 complex subunit 2, mitochondrial OS=Homo sapiens GN=UQCRC2 PE=1 SV=3 - [QCR2_HUMAN]	0.00265 5378	1.24236 4634
P31930	Cytochrome b-c1 complex subunit 1, mitochondrial OS=Homo sapiens GN=UQCRC1 PE=1 SV=3 - [QCR1_HUMAN]	0.00000 024	1.34378 4994
P52943	Cysteine-rich protein 2 OS=Homo sapiens GN=CRIP2 PE=1 SV=1 - [CRIP2_HUMAN]	0.04196 6923	0.78786 6851
Q16527	Cysteine and glycine-rich protein 2 OS=Homo sapiens GN=CSRP2 PE=1 SV=3 - [CSRP2_HUMAN]	2.77E-0 9	0.76574 5645
P04080	Cystatin-B OS=Homo sapiens GN=CSTB PE=1 SV=2 - [CYTB_HUMAN]	0.04042 3221	0.36525 756
P42771	Cyclin-dependent kinase inhibitor 2A OS=Homo sapiens GN=CDKN2A PE=1 SV=2 - [CDN2A_HUMAN]	0.02792 2354	0.60694 9573
Q9ULV4	Coronin-1C OS=Homo sapiens GN=CORO1C PE=1 SV=1 - [COR1C_HUMAN]	8.47E-1 7	0.74287 7465
Q9BR76	Coronin-1B OS=Homo sapiens GN=CORO1B PE=1 SV=1 - [COR1B_HUMAN]	0.00000 609	0.72707 4434
O75131	Copine-3 OS=Homo sapiens GN=CPNE3 PE=1 SV=1 - [CPNE3_HUMAN]	1.53E-0 9	1.23272 8954
P02748	Complement component C9 OS=Homo sapiens GN=C9 PE=1	1.96E-4	2.21781

	SV=2 - [CO9_HUMAN]	8	712
P07360	Complement component C8 gamma chain OS=Homo sapiens GN=C8G PE=1 SV=3 - [CO8G_HUMAN]	1.05E-14	1.999454693
P07358	Complement component C8 beta chain OS=Homo sapiens GN=C8B PE=1 SV=3 - [CO8B_HUMAN]	2.98E-26	1.95588395
P07357	Complement component C8 alpha chain OS=Homo sapiens GN=C8A PE=1 SV=2 - [CO8A_HUMAN]	1.47E-15	2.006566948
P10643	Complement component C7 OS=Homo sapiens GN=C7 PE=1 SV=2 - [CO7_HUMAN]	2.62E-45	2.059523809
P13671	Complement component C6 OS=Homo sapiens GN=C6 PE=1 SV=3 - [CO6_HUMAN]	3.88E-30	2.184537914
Q07021	Complement component 1 Q subcomponent-binding protein, mitochondrial OS=Homo sapiens GN=C1QBP PE=1 SV=1 - [C1QBP_HUMAN]	0.0000811	1.240226476
P01031	Complement C5 OS=Homo sapiens GN=C5 PE=1 SV=4 - [CO5_HUMAN]	1.76E-66	2.290043662
P01024	Complement C3 OS=Homo sapiens GN=C3 PE=1 SV=2 - [CO3_HUMAN]	8.41E-55	1.589222988
P12111	Collagen alpha-3(VI) chain OS=Homo sapiens GN=COL6A3 PE=1 SV=5 - [CO6A3_HUMAN]	9.32E-24	1.740599483
P12109	Collagen alpha-1(VI) chain OS=Homo sapiens GN=COL6A1 PE=1 SV=3 - [CO6A1_HUMAN]	0.009817161	1.566170713
P23528	Cofilin-1 OS=Homo sapiens GN=CFL1 PE=1 SV=3 - [COF1_HUMAN]	2.97E-43	0.679842665
P48444	Coatomer subunit delta OS=Homo sapiens GN=ARCN1 PE=1 SV=1 - [COPD_HUMAN]	0.000347719	0.79928679
P12259	Coagulation factor V OS=Homo sapiens GN=F5 PE=1 SV=4 - [FA5_HUMAN]	0.000000192	1.449065353
Q14019	Coactosin-like protein OS=Homo sapiens GN=COTL1 PE=1 SV=3 - [COTL1_HUMAN]	2E-32	0.656311529
P10909	Clusterin OS=Homo sapiens GN=CLU PE=1 SV=1 - [CLUS_HUMAN]	3.78E-56	2.142923693
P09497	Clathrin light chain B OS=Homo sapiens GN=CLTB PE=1 SV=1 - [CLCB_HUMAN]	1.97E-08	0.731172313
P09496	Clathrin light chain A OS=Homo sapiens GN=CLTA PE=1 SV=1 - [CLCA_HUMAN]	0.0000222	0.710900474
Q13185	Chromobox protein homolog 3 OS=Homo sapiens GN=CBX3 PE=1 SV=4 - [CBX3_HUMAN]	0.000991485	0.769484445
Q96NY7	Chloride intracellular channel protein 6 OS=Homo sapiens GN=CLIC6 PE=2 SV=3 - [CLIC6_HUMAN]	6.92E-10	0.794377572
Q9Y696	Chloride intracellular channel protein 4 OS=Homo sapiens GN=CLIC4 PE=1 SV=4 - [CLIC4_HUMAN]	4.81E-28	0.749830729

P00450	Ceruloplasmin OS=Homo sapiens GN=CP PE=1 SV=1 - [CERU_HUMAN]	0.000519804	1.550387597
P62633	Cellular nucleic acid-binding protein OS=Homo sapiens GN=CNBP PE=1 SV=1 - [CNBP_HUMAN]	0.034310407	0.769082869
Q8NFZ8	Cell adhesion molecule 4 OS=Homo sapiens GN=CADM4 PE=1 SV=1 - [CADM4_HUMAN]	0.035023488	0.741152492
Q9BY67	Cell adhesion molecule 1 OS=Homo sapiens GN=CADM1 PE=1 SV=2 - [CADM1_HUMAN]	0.031440789	0.790513834
Q9NZ45	CDGSH iron-sulfur domain-containing protein 1 OS=Homo sapiens GN=CISD1 PE=1 SV=1 - [CISD1_HUMAN]	0.000119289	1.320132013
P21926	CD9 antigen OS=Homo sapiens GN=CD9 PE=1 SV=4 - [CD9_HUMAN]	0.002016495	1.427297056
Q13740	CD166 antigen OS=Homo sapiens GN=ALCAM PE=1 SV=2 - [CD166_HUMAN]	1.43E-09	0.761503565
P07339	Cathepsin D OS=Homo sapiens GN=CTSD PE=1 SV=1 - [CATD_HUMAN]	2.33E-41	0.397204951
P07858	Cathepsin B OS=Homo sapiens GN=CTSB PE=1 SV=3 - [CATB_HUMAN]	2.99E-15	0.671293799
P35222	Catenin beta-1 OS=Homo sapiens GN=CTNNB1 PE=1 SV=1 - [CTNB1_HUMAN]	0.00000587	0.739936859
P35221	Catenin alpha-1 OS=Homo sapiens GN=CTNNA1 PE=1 SV=1 - [CTNA1_HUMAN]	7.01E-10	0.78597982
O75718	Cartilage-associated protein OS=Homo sapiens GN=CRTAP PE=1 SV=1 - [CRTAP_HUMAN]	0.00000112	0.775241875
Q9UI42	Carboxypeptidase A4 OS=Homo sapiens GN=CPA4 PE=1 SV=2 - [CBPA4_HUMAN]	0.000168639	0.691195236
O43852	Calumenin OS=Homo sapiens GN=CALU PE=1 SV=2 - [CALU_HUMAN]	1.18E-30	0.715492398
P27797	Calreticulin OS=Homo sapiens GN=CALR PE=1 SV=1 - [CALR_HUMAN]	3.75E-69	0.714003078
Q15417	Calponin-3 OS=Homo sapiens GN=CNN3 PE=1 SV=1 - [CNN3_HUMAN]	5.74E-47	0.566285828
Q99439	Calponin-2 OS=Homo sapiens GN=CNN2 PE=1 SV=4 - [CNN2_HUMAN]	2.36E-18	0.691374042
P51911	Calponin-1 OS=Homo sapiens GN=CNN1 PE=1 SV=2 - [CNN1_HUMAN]	1.32E-28	0.554133443
P62158	Calmodulin OS=Homo sapiens GN=CALM1 PE=1 SV=2 - [CALM_HUMAN]	2.32E-31	0.663504351
Q05682	Caldesmon OS=Homo sapiens GN=CALD1 PE=1 SV=3 - [CALD1_HUMAN]	9.66E-100	0.451011582
Q6NUK1	Calcium-binding mitochondrial carrier protein SCaMC-1 OS=Homo sapiens GN=SLC25A24 PE=1 SV=2 -	0.000471249	1.23583934

	[SCMC1_HUMAN]		
Q9UJS0	Calcium-binding mitochondrial carrier protein Aralar2 OS=Homo sapiens GN=SLC25A13 PE=1 SV=2 - [CMC2_HUMAN]	0.00000024	1.450083658
P19022	Cadherin-2 OS=Homo sapiens GN=CDH2 PE=1 SV=4 - [CADH2_HUMAN]	0.013946789	0.744720464
P04003	C4b-binding protein alpha chain OS=Homo sapiens GN=C4BPA PE=1 SV=2 - [C4BPA_HUMAN]	0.006432456	1.683029452
Q9H8M2	Bromodomain-containing protein 9 OS=Homo sapiens GN=BRD9 PE=1 SV=2 - [BRD9_HUMAN]	0.0000339	2.215657313
P54687	Branched-chain-amino-acid aminotransferase, cytosolic OS=Homo sapiens GN=BCAT1 PE=1 SV=3 - [BCAT1_HUMAN]	0.000220854	0.719262756
P80723	Brain acid soluble protein 1 OS=Homo sapiens GN=BASP1 PE=1 SV=2 - [BASP1_HUMAN]	3.1E-11	0.492676431
P07738	Bisphosphoglycerate mutase OS=Homo sapiens GN=BPGM PE=1 SV=2 - [PMGE_HUMAN]	0.001688146	1.324942034
P21810	Biglycan OS=Homo sapiens GN=BGN PE=1 SV=2 - [PGS1_HUMAN]	0.018733958	1.329875657
O95340	Bifunctional 3'-phosphoadenosine 5'-phosphosulfate synthase 2 OS=Homo sapiens GN=PAPSS2 PE=1 SV=2 - [PAPS2_HUMAN]	2.24E-08	1.219140506
P07686	Beta-hexosaminidase subunit beta OS=Homo sapiens GN=HEXB PE=1 SV=3 - [HEXB_HUMAN]	0.000000068	0.785896726
P06865	Beta-hexosaminidase subunit alpha OS=Homo sapiens GN=HEXA PE=1 SV=2 - [HEXA_HUMAN]	0.0000516	0.67347626
P16278	Beta-galactosidase OS=Homo sapiens GN=GLB1 PE=1 SV=2 - [BGAL_HUMAN]	0.000110769	0.774365408
Q562R1	Beta-actin-like protein 2 OS=Homo sapiens GN=ACTBL2 PE=1 SV=2 - [ACTBL_HUMAN]	0.048964546	0.647144475
Q96BJ3	Axin interactor, dorsalization-associated protein OS=Homo sapiens GN=AIDA PE=1 SV=1 - [AIDA_HUMAN]	0.000363321	0.743080067
Q9UII2	ATPase inhibitor, mitochondrial OS=Homo sapiens GN=ATPIF1 PE=1 SV=1 - [ATIF1_HUMAN]	2.79E-18	0.560437141
Q9NVI7	ATPase family AAA domain-containing protein 3A OS=Homo sapiens GN=ATAD3A PE=1 SV=2 - [ATD3A_HUMAN]	0.00000612	1.211974306
P48047	ATP synthase subunit O, mitochondrial OS=Homo sapiens GN=ATP5O PE=1 SV=1 - [ATPO_HUMAN]	0.0000955	1.300340383
P36542	ATP synthase subunit gamma, mitochondrial OS=Homo sapiens GN=ATP5C1 PE=1 SV=1 - [ATPG_HUMAN]	0.002764607	1.245512492
O75964	ATP synthase subunit g, mitochondrial OS=Homo sapiens GN=ATP5L PE=1 SV=3 - [ATP5L_HUMAN]	1.64E-13	1.467471059
P56134	ATP synthase subunit f, mitochondrial OS=Homo sapiens	0.00000	1.33856

	GN=ATP5J2 PE=1 SV=3 - [ATPK_HUMAN]	0041	0091
P56385	ATP synthase subunit e, mitochondrial OS=Homo sapiens GN=ATP5I PE=1 SV=2 - [ATP5I_HUMAN]	8.74E-08	1.30818707
O75947	ATP synthase subunit d, mitochondrial OS=Homo sapiens GN=ATP5H PE=1 SV=3 - [ATP5H_HUMAN]	1.74E-11	1.47368421
P25705	ATP synthase subunit alpha, mitochondrial OS=Homo sapiens GN=ATP5A1 PE=1 SV=1 - [ATPA_HUMAN]	4.18E-40	1.267958378
P24539	ATP synthase F(0) complex subunit B1, mitochondrial OS=Homo sapiens GN=ATP5F1 PE=1 SV=2 - [AT5F1_HUMAN]	0.000000446	1.219413055
Q12797	Aspartyl/asparaginyl beta-hydroxylase OS=Homo sapiens GN=ASPH PE=1 SV=3 - [ASPH_HUMAN]	0.0000123	1.213327186
P02649	Apolipoprotein E OS=Homo sapiens GN=APOE PE=1 SV=1 - [APOE_HUMAN]	9.66E-16	1.823078518
P04114	Apolipoprotein B-100 OS=Homo sapiens GN=APOB PE=1 SV=2 - [APOB_HUMAN]	0.000167731	1.456001456
P06727	Apolipoprotein A-IV OS=Homo sapiens GN=APOA4 PE=1 SV=3 - [APOA4_HUMAN]	3.82E-24	1.875234403
P02647	Apolipoprotein A-I OS=Homo sapiens GN=APOA1 PE=1 SV=1 - [APOA1_HUMAN]	9.52E-12	1.867716921
P61966	AP-1 complex subunit sigma-1A OS=Homo sapiens GN=AP1S1 PE=1 SV=1 - [AP1S1_HUMAN]	0.048358688	1.347406242
P01008	Antithrombin-III OS=Homo sapiens GN=SERPINC1 PE=1 SV=1 - [ANT3_HUMAN]	4.62E-14	1.596458763
P08758	Annexin A5 OS=Homo sapiens GN=ANXA5 PE=1 SV=2 - [ANXA5_HUMAN]	3.13E-11	0.694470002
P07355	Annexin A2 OS=Homo sapiens GN=ANXA2 PE=1 SV=2 - [ANXA2_HUMAN]	3.27E-46	0.593176957
Q9BY76	Angiopoietin-related protein 4 OS=Homo sapiens GN=ANGPTL4 PE=1 SV=2 - [ANGL4_HUMAN]	3.22E-11	1.779266314
Q9NVD7	Alpha-parvin OS=Homo sapiens GN=PARVA PE=1 SV=1 - [PARVA_HUMAN]	0.0409168	0.775013563
P06733	Alpha-enolase OS=Homo sapiens GN=ENO1 PE=1 SV=2 - [ENOA_HUMAN]	4.15E-142	0.739407026
O43707	Alpha-actinin-4 OS=Homo sapiens GN=ACTN4 PE=1 SV=2 - [ACTN4_HUMAN]	2.16E-76	0.442345811
P12814	Alpha-actinin-1 OS=Homo sapiens GN=ACTN1 PE=1 SV=2 - [ACTN1_HUMAN]	6.22E-134	0.581066026
P30533	Alpha-2-macroglobulin receptor-associated protein OS=Homo sapiens GN=LRPAP1 PE=1 SV=1 - [AMRP_HUMAN]	5.24E-09	0.697337273
P05091	Aldehyde dehydrogenase, mitochondrial OS=Homo sapiens GN=ALDH2 PE=1 SV=2 - [ALDH2_HUMAN]	0.0000452	1.365187713
O00468	Agrin OS=Homo sapiens GN=AGRN PE=1 SV=5 -	0.03625	0.74844

	[AGRIN_HUMAN]	314	2971
Q9Y4W6	AFG3-like protein 2 OS=Homo sapiens GN=AFG3L2 PE=1 SV=2 - [AFG32_HUMAN]	0.01895 9304	1.29407 9586
P12236	ADP/ATP translocase 3 OS=Homo sapiens GN=SLC25A6 PE=1 SV=4 - [ADT3_HUMAN]	4.78E-0 9	1.26156 4339
Q01518	Adenylyl cyclase-associated protein 1 OS=Homo sapiens GN=CAP1 PE=1 SV=5 - [CAP1_HUMAN]	3.78E-1 5	0.41087 553
P30566	Adenylosuccinate lyase OS=Homo sapiens GN=ADSL PE=1 SV=2 - [PUR8_HUMAN]	0.00006 96	0.79317 8663
O15511	Actin-related protein 2/3 complex subunit 5 OS=Homo sapiens GN=ARPC5 PE=1 SV=3 - [ARPC5_HUMAN]	0.00092 9186	0.79264 1643
P59998	Actin-related protein 2/3 complex subunit 4 OS=Homo sapiens GN=ARPC4 PE=1 SV=3 - [ARPC4_HUMAN]	0.00005 98	0.78084 7376
O15144	Actin-related protein 2/3 complex subunit 2 OS=Homo sapiens GN=ARPC2 PE=1 SV=1 - [ARPC2_HUMAN]	1.99E-1 1	0.77651 0556
P60709	Actin, cytoplasmic 1 OS=Homo sapiens GN=ACTB PE=1 SV=1 - [ACTB_HUMAN]	6.58E-2 43	0.57642 321
P62736	Actin, aortic smooth muscle OS=Homo sapiens GN=ACTA2 PE=1 SV=1 - [ACTA_HUMAN]	0.00001 41	0.69349 7307
P39687	Acidic leucine-rich nuclear phosphoprotein 32 family member A OS=Homo sapiens GN=ANP32A PE=1 SV=1 - [AN32A_HUMAN]	0.00020 6454	0.66401 0624
Q9BWD1	Acetyl-CoA acetyltransferase, cytosolic OS=Homo sapiens GN=ACAT2 PE=1 SV=2 - [THIC_HUMAN]	0.00568 6991	0.77002 0534
A1L0T0	Acetolactate synthase-like protein OS=Homo sapiens GN=ILVBL PE=1 SV=2 - [ILVBL_HUMAN]	0.01243 0828	1.23250 2861
P36578	60S ribosomal protein L4 OS=Homo sapiens GN=RPL4 PE=1 SV=5 - [RL4_HUMAN]	0.00005 14	0.79039 4821
P63173	60S ribosomal protein L38 OS=Homo sapiens GN=RPL38 PE=1 SV=2 - [RL38_HUMAN]	4.24E-0 8	1.22376 5527
P61513	60S ribosomal protein L37a OS=Homo sapiens GN=RPL37A PE=1 SV=2 - [RL37A_HUMAN]	0.01389 5573	0.79621 004
Q9Y3U8	60S ribosomal protein L36 OS=Homo sapiens GN=RPL36 PE=1 SV=3 - [RL36_HUMAN]	0.00115 5801	0.74973 7592
P47914	60S ribosomal protein L29 OS=Homo sapiens GN=RPL29 PE=1 SV=2 - [RL29_HUMAN]	0.00385 6154	0.67189 2497
P61353	60S ribosomal protein L27 OS=Homo sapiens GN=RPL27 PE=1 SV=2 - [RL27_HUMAN]	4.43E-1 2	0.78971 1192
P62750	60S ribosomal protein L23a OS=Homo sapiens GN=RPL23A PE=1 SV=1 - [RL23A_HUMAN]	2.4E-11	0.71884 058
P35268	60S ribosomal protein L22 OS=Homo sapiens GN=RPL22 PE=1 SV=2 - [RL22_HUMAN]	0.00000 0358	0.69534 4353

P27635	60S ribosomal protein L10 OS=Homo sapiens GN=RPL10 PE=1 SV=4 - [RL10_HUMAN]	0.026797114	0.778351451
P05386	60S acidic ribosomal protein P1 OS=Homo sapiens GN=RPLP1 PE=1 SV=1 - [RLA1_HUMAN]	0.005729577	0.771522256
P05388	60S acidic ribosomal protein P0 OS=Homo sapiens GN=RPLP0 PE=1 SV=1 - [RLA0_HUMAN]	0.000000427	0.772929402
P21589	5'-nucleotidase OS=Homo sapiens GN=NT5E PE=1 SV=1 - [5NTD_HUMAN]	1.23E-16	1.480154248
P08195	4F2 cell-surface antigen heavy chain OS=Homo sapiens GN=SLC3A2 PE=1 SV=3 - [4F2_HUMAN]	1.6E-15	1.340350463
P62861	40S ribosomal protein S30 OS=Homo sapiens GN=FAU PE=1 SV=1 - [RS30_HUMAN]	0.000084	0.718519849
P62857	40S ribosomal protein S28 OS=Homo sapiens GN=RPS28 PE=1 SV=1 - [RS28_HUMAN]	0.000226711	0.387847447
P39019	40S ribosomal protein S19 OS=Homo sapiens GN=RPS19 PE=1 SV=2 - [RS19_HUMAN]	1.02E-23	0.665710774
P25398	40S ribosomal protein S12 OS=Homo sapiens GN=RPS12 PE=1 SV=3 - [RS12_HUMAN]	4.19E-12	0.764266305
Q02218	2-oxoglutarate dehydrogenase, mitochondrial OS=Homo sapiens GN=OGDH PE=1 SV=3 - [ODO1_HUMAN]	0.00004	1.247660636
Q13442	28 kDa heat- and acid-stable phosphoprotein OS=Homo sapiens GN=PDAP1 PE=1 SV=1 - [HAP28_HUMAN]	0.000376469	0.673238759
Q16698	2,4-dienoyl-CoA reductase, mitochondrial OS=Homo sapiens GN=DECR1 PE=1 SV=1 - [DECR_HUMAN]	0.000225727	1.301115241
Q15147	1-phosphatidylinositol 4,5-bisphosphate phosphodiesterase beta-4 OS=Homo sapiens GN=PLCB4 PE=1 SV=3 - [PLCB4_HUMAN]	0.006859749	0.563261283
P63104	14-3-3 protein zeta/delta OS=Homo sapiens GN=YWHAZ PE=1 SV=1 - [1433Z_HUMAN]	9.66E-36	0.696780394
P27348	14-3-3 protein theta OS=Homo sapiens GN=YWHAQ PE=1 SV=1 - [1433T_HUMAN]	2.09E-10	0.6722438
P61981	14-3-3 protein gamma OS=Homo sapiens GN=YWHAG PE=1 SV=2 - [1433G_HUMAN]	4.08E-14	0.75666473
P62258	14-3-3 protein epsilon OS=Homo sapiens GN=YWHAE PE=1 SV=1 - [1433E_HUMAN]	7.43E-29	0.748948663
P31946	14-3-3 protein beta/alpha OS=Homo sapiens GN=YWHAB PE=1 SV=3 - [1433B_HUMAN]	0.005560073	0.659558096

Table S2 Differentially expressed proteins in low risk group after smoker serum stimulation (LS vs LN)(ratio<0.8 or ratio>1.2, P<0.05)

Accession	Description	p_value	ratio
O00192	Armadillo repeat protein deleted in velo-cardio-facial syndrome OS=Homo sapiens GN=ARVCF PE=1 SV=1 - [ARVC_HUMAN]	0.0000149	0.759734093
O95857	Tetraspanin-13 OS=Homo sapiens GN=TSPAN13 PE=2 SV=1 - [TSN13_HUMAN]	0.0000598	0.692640693
P01009	Alpha-1-antitrypsin OS=Homo sapiens GN=SERPINA1 PE=1 SV=3 - [A1AT_HUMAN]	0.00000506	1.256369094
P01742	Ig heavy chain V-I region EU OS=Homo sapiens PE=1 SV=1 - [HV101_HUMAN]	0.000641665	1.474722337
P01781	Ig heavy chain V-III region GAL OS=Homo sapiens PE=1 SV=1 - [HV320_HUMAN]	0.002081654	1.226492233
P02647	Apolipoprotein A-I OS=Homo sapiens GN=APOA1 PE=1 SV=1 - [APOA1_HUMAN]	0.000000233	1.212057336
P02671	Fibrinogen alpha chain OS=Homo sapiens GN=FGA PE=1 SV=2 - [FIBA_HUMAN]	3.13E-11	1.354797577
P02675	Fibrinogen beta chain OS=Homo sapiens GN=FGB PE=1 SV=2 - [FIBB_HUMAN]	0.004339253	1.309669728
P02679	Fibrinogen gamma chain OS=Homo sapiens GN=FGG PE=1 SV=3 - [FIBG_HUMAN]	3.98E-14	1.397077314
P02795	Metallothionein-2 OS=Homo sapiens GN=MT2A PE=1 SV=1 - [MT2_HUMAN]	0.00025278	1.460446248
P05161	Ubiquitin-like protein ISG15 OS=Homo sapiens GN=ISG15 PE=1 SV=5 - [ISG15_HUMAN]	0.01782316	1.428911646
P05362	Intercellular adhesion molecule 1 OS=Homo sapiens GN=ICAM1 PE=1 SV=2 - [ICAM1_HUMAN]	0.001066494	1.22294774
P07357	Complement component C8 alpha chain OS=Homo sapiens GN=C8A PE=1 SV=2 - [CO8A_HUMAN]	0.000460832	1.204731308
P09493	Tropomyosin alpha-1 chain OS=Homo sapiens GN=TPM1 PE=1 SV=2 - [TPM1_HUMAN]	0.000000119	0.79636256
P09601	Heme oxygenase 1 OS=Homo sapiens GN=HMOX1 PE=1 SV=1 - [HMOX1_HUMAN]	0.01229211	1.419950302
P09914	Interferon-induced protein with tetratricopeptide repeats 1 OS=Homo sapiens GN=IFIT1 PE=1 SV=2 - [IFIT1_HUMAN]	0.013631126	1.895221335
P10909	Clusterin OS=Homo sapiens GN=CLU PE=1 SV=1 - [CLUS_HUMAN]	0.0000043	1.22097106
P13671	Complement component C6 OS=Homo sapiens GN=C6 PE=1 SV=3 - [CO6_HUMAN]	0.000142431	1.205853498

P20591	Interferon-induced GTP-binding protein Mx1 OS=Homo sapiens GN=MX1 PE=1 SV=4 - [MX1_HUMAN]	0.03179 8016	2.2463 4968
P42224	Signal transducer and activator of transcription 1-alpha/beta OS=Homo sapiens GN=STAT1 PE=1 SV=2 - [STAT1_HUMAN]	0.01677 8016	1.2005 40244
P43490	Nicotinamide phosphoribosyltransferase OS=Homo sapiens GN=NAMPT PE=1 SV=1 - [NAMPT_HUMAN]	0.00001 34	1.2978 58533
P56377	AP-1 complex subunit sigma-2 OS=Homo sapiens GN=AP1S2 PE=1 SV=1 - [AP1S2_HUMAN]	0.01559 6663	0.6500 89387
P62328	Thymosin beta-4 OS=Homo sapiens GN=TMSB4X PE=1 SV=2 - [TYB4_HUMAN]	0.00000 0232	1.2259 91009
Q16363	Laminin subunit alpha-4 OS=Homo sapiens GN=LAMA4 PE=1 SV=4 - [LAMA4_HUMAN]	0.00082 4632	0.7632 61671
Q6UX71	Plexin domain-containing protein 2 OS=Homo sapiens GN=PLXDC2 PE=1 SV=1 - [PXDC2_HUMAN]	0.00530 3444	0.7574 80116
Q9BYC5	Alpha-(1,6)-fucosyltransferase OS=Homo sapiens GN=FUT8 PE=1 SV=2 - [FUT8_HUMAN]	0.00103 6418	1.2511 72975
Q9H3P7	Golgi resident protein GCP60 OS=Homo sapiens GN=ACBD3 PE=1 SV=4 - [GCP60_HUMAN]	0.04075 7832	0.6944 44444
Q9NZR1	Tropomodulin-2 OS=Homo sapiens GN=TMOD2 PE=1 SV=1 - [TMOD2_HUMAN]	0.04012 3839	0.7209 80534
Q9UL46	Proteasome activator complex subunit 2 OS=Homo sapiens GN=PSME2 PE=1 SV=4 - [PSME2_HUMAN]	0.00195 3518	1.2204 4241
Q9Y4W6	AFG3-like protein 2 OS=Homo sapiens GN=AFG3L2 PE=1 SV=2 - [AFG32_HUMAN]	0.00032 018	0.6999 12511
